# Effect of Shenkang on renal fibrosis and activation of renal interstitial fibroblasts through the JAK2/STAT3 pathway

**DOI:** 10.1186/s12906-020-03180-3

**Published:** 2021-01-06

**Authors:** Tianyu Qin, You Wu, Tonghua Liu, Lili Wu

**Affiliations:** 1 Beijing University of Chinese Medicine, Beijing, 100029 China; 2 Key Laboratory of Health Cultivation of the Ministry of Education, Beijing University of Chinese Medicine, Beijing, 100029 China

**Keywords:** Shenkang, Chronic kidney disease, Renal fibrosis, UUO, Renal fibroblast activation, JAK2/STAT3 pathway

## Abstract

**Background:**

Activation of renal fibroblasts is a critical mechanism in the process of renal fibrosis. As a commonly used herbal formula, Shenkang (SK) has been found to attenuate renal fibrosis and renal parenchyma destruction. However, the effect of SK on renal fibroblast activation in unilateral ureteral obstruction (UUO) mice and its molecular mechanism remain undetermined. The present study was performed to elucidate the effect of SK on renal fibroblast activation and renal fibrosis, as well as the potential underlying mechanism, in both NRK-49F cells and UUO mice.

**Methods:**

NRK-49F cells were stimulated with 10 ng/ml TGF-β1 for 48 h. After SK treatment, the CCK-8 method was used to evaluate cell viability. Thirty-six C57BL/6 mice were randomly divided into the sham group, UUO group, angiotensin receptor blocker (ARB) group, and SK high-, moderate- and low-dose groups. UUO was induced in mice except those in the sham group. Drugs were administered 1 day later. On the 13th day, the fractional anisotropy (FA) value was determined by MRI to evaluate the degree of renal fibrosis. After 14 days, serum indexes were assessed. Hematoxylin and eosin (HE) and Sirius red staining were used to observe pathological morphology and the degree of fibrosis of the affected kidney. Western blotting and PCR were used to assess the expression of related molecules in both cells and animals at the protein and gene levels.

**Results:**

Our results showed that SK reduced extracellular matrix (ECM) and α-smooth muscle actin (α-SMA) expression both in vitro and in vivo and attenuated renal fibrosis and the pathological lesion degree after UUO, suppressing JAK2/STAT3 activation. Furthermore, we found that SK regulated the JAK2/STAT3 pathway regulators peroxiredoxin 5 (Prdx5) in vitro and suppressor of cytokine signaling protein 1 (SOCS1) and SOCS3 in vivo.

**Conclusions:**

These results indicated that SK inhibited fibroblast activation by regulating the JAK2/STAT3 pathway, which may be a mechanism underlying its protective action in renal fibrosis.

## Background

Chronic kidney disease (CKD) results from a variety of different diseases that irreversibly damage the kidney and cause various types of complications in patients [1]. For patients undergoing dialysis, quality of life is greatly affected, and medical costs are very high [2]. The primary pathological process of renal injury leads to normal renal parenchyma destruction and progressive scar tissue formation, which ultimately leads to fibrosis. Renal fibrosis includes tubular interstitial fibrosis and glomerulosclerosis [3], leading to the destruction of renal tissue and loss of function. Renal tubulointerstitial fibrosis is the typical pathway for the progressive development of almost all CKDs and the main pathological basis for end-stage renal disease, which is characterized by tubular epithelial cell atrophy, inflammatory cell infiltration, aberrant activation and growth of renal fibroblasts and excessive extracellular matrix (ECM) accumulation [4, 5]. The effector cells, α-smooth muscle actin (α-SMA)-positive myofibroblasts, synthesize and secrete ECM. The activation of interstitial fibroblasts into myofibroblasts could help repair damaged tissue. However, when this repair process is abnormal and excessive ECM is secreted, irreversible kidney damage results. Accordingly, the signaling pathway that mediates myofibroblast activation may be a target for alleviating the process of renal fibrosis.

The Janus kinase/signal transducer and activator of transcription (JAK/STAT) pathway is a pleiotropic signaling cascade for multiple growth factors and cytokines [6] that mediates a variety of cellular functions, including cell survival and proliferation [7]. STAT3 is activated by tyrosine (Tyr) phosphorylation at Tyr705 through Janus kinase in response to a variety of growth factors and cytokines, including transforming growth factor β (TGF-β) [8]. Phosphorylated STAT3 forms dimers, which are then transferred to the nucleus, where they directly bind to the DNA sequence and regulate the expression of target genes [9]. The activation of STAT3 and JAK2 is increased in fibrogenic renal interstitial fibroblasts induced by unilateral ureteral obstruction (UUO) [10, 11]. An increase in STAT3 phosphorylation has also been observed in TGF-β-treated NRK-49F cells [12].

Current widely used treatments for CKD are often accompanied by less than satisfactory effects [13] and common side effects. Thus, it is of great significance to investigate and identify effective treatment measures to prevent and control the occurrence and development of CKD to improve the prognosis of patients. Shenkang (SK) is a commonly used herbal formula that contains rhubarb (*Rheum palmatum* L. or *R. tanguticum* Maxim. ex Balf.), red sage (*Salvia miltiorrhiza* Bunge), safflower (*Carthamus tinctorius* L.), and astragalus (*Astragalus mongholicus* Bunge). Since the 1990s, SK has been used in China to treat CKD and related diseases, including diabetic nephropathy (DN), chronic renal failure, glomerulonephritis, chronic nephritis and renal insufficiency. With its obvious therapeutic effects and few side effects, SK may delay the progression of renal dysfunction in CKD. In a clinical trial of 2200 people, the efficacy of SK in protecting against chronic renal failure and symptoms associated with CKD following treatment with traditional Chinese medicine was 73.05 and 98.00%, respectively. Additionally, the creatinine clearance rate and serum creatinine (SCr) level remained stable, showing that SK has good efficacy and is safe for the treatment of CKD [14]. It has been verified that SK is able to alleviate CKD and renal fibrosis by mitigating fibrosis, inflammation [15] and apoptosis [16]. However, most of the existing studies on the subject were not sufficiently comprehensive and mainly focused on TGF-β and related pathways. The UUO model is acknowledged as an experimental model for investigating renal fibrosis. In the present study, we studied the effects of SK on NRK-49F fibroblast cell activation and renal fibrosis in UUO mice. We further demonstrated that these curative effects of SK may have been achieved by targeting the JAK2/STAT3 pathway in cultured NRK-49F cells and a UUO mouse model.

## Methods

### Chemicals and reagents

SK was purchased from Shijishenkang Pharmaceutical Company, Ltd. (Xi’an, China). Losartan potassium tablets were purchased from Merck Sharp & Dohme Pharmaceutical Company, Ltd. (Hangzhou, China).

### Cell culture

The rat kidney fibroblast cell line (NRK-49F) was obtained from the American Type Cell Collection (ATCC, Manassas, VA, USA). NRK-49F cells were cultured in complete medium, i.e., Dulbecco’s modified Eagle’s medium (Invitrogen, CA, USA) containing 5% fetal bovine serum, 1% penicillin and streptomycin in an atmosphere of 5% CO_2_ and 95% air at 37 °C. Then, the cells were treated with or without TGF-β1 (10 ng/mL) (Novoprotein, Shanghai, China), an angiotensin receptor blocker (ARB) (1 mg/mL) and gradient concentrations of SK following adherence, and cell viability was evaluated.

### Assays of cell viability

The viability of NRK-49F cells was determined using the Cell Counting Kit-8 (Dojindo, Kyushu, Japan) according to the kit’s instructions. NRK-49F cells were plated at a density of 5 × 10^4^ cells per well in 96-well plates. There were 5 replicates for each treatment. After 24 h or 48 h of culture, the medium in each well was replaced with 100 μL of serum-free medium containing CCK-8 (10:1), and the cells were incubated in an incubator for another 2 h. The absorbance was measured at a wavelength of 450 nm. The effect of treatment was calculated as the percentage of living cells relative to living control cells treated with vehicle only.

### Animals

The experimental procedures used in this study were approved by the Animal Care and Ethics Committee of Beijing University of Traditional Chinese Medicine (No. BUCM-4-2,018,060,419-2023) and conformed to internationally recognized principles of laboratory animal use and care. Thirty-six 8-week-old C57BL/6 male mice were purchased from Beijing Vital River Laboratory Animal Technology Co., Ltd. (SYXK (Jing), 2017–0033). The mice were placed in a special pathogen-free animal laboratory in an air-conditioned room (light: 12-h light/dark cycle; room temperature: 25 ± 1 °C; relative humidity: 50 ± 10%) and randomly divided into the following six groups: the sham group, the UUO group, the ARB group and the high-, moderate-, and low-dose SK groups. UUO was induced in the mice in the UUO group, the ARB group and the high-, moderate-, and low-dose SK groups according to a previously established procedure. Anesthesia was induced with 2% isoflurane and maintained with 1.5% isoflurane. A 1- to 2-cm longitudinal incision was made in the left midabdomen, and the left ureter was separated bluntly, ligated at two places and then cut between the two ligations. Then, the abdominal cavity was closed, after which analgesia was given for 3 days. The mice in the sham group underwent similar surgical procedures, including pre- and postoperative anesthesia, laparotomy, and blunt separation of the ureter, without ureteral ligation and cutting. From the second day after obstruction, losartan was intragastrically administered to the mice in the ARB group at a dose of 0.13 mg/10 g (0.15 mL/10 g). SK at a high dose of 0.08 g/10 g (0.13 mL/10 g), a moderate dose of 0.04 g/10 g (0.13 mL/10 g), or a low dose of 0.02 g/10 g (0.13 mL/10 g) was given to mice in the SK group via the tail vein for 14 days after ureteral ligation. Saline (0.13 mL/10 g) was given via the tail vein to the mice in the sham, UUO and ARB groups, and saline (0.15 mL/10 g) was given via gavage to the mice in the sham, UUO and SK groups to reduce bias. After MRI, blood was collected from the mice by cardiac puncture upon sacrifice under deep anesthesia (induced and maintained with 2% isoflurane) on the 14th day, and kidney tissues were collected. Serum was obtained from whole blood by centrifugation at 1000 (× g) (4 °C) for 10 min. Serum blood urea nitrogen (BUN), SCr and cystatin C (Cys-C) levels were measured by the colorimetric method. Hematoxylin and eosin (HE) staining and Sirius red staining were performed for the detection of histopathology and collagen in stromal structures of the affected kidney, respectively. For HE staining, the slices were soaked in distilled water after dewaxing, soaked in hematoxylin staining solution (Solarbio Science & Technology, China) followed by 1% hydrochloric acid alcohol solution for color separation, washed with tap water, turned blue, and stained with eosin dye. The stained slices were washed with distilled water, dehydrated, cleared, and sealed with neutral gum. For Sirius red staining, the slices were dewaxed, stained with Harris hematoxylin, washed, stained with Sirius red (Solarbio Science & Technology, China), directly separated and dehydrated with absolute ethanol, cleared with xylene, and sealed. Five visual fields of the renal cortex and proximal tubule area were randomly selected for each mouse. ImageJ software was used to calculate the red area, and the average red area was calculated to determine the Sirius red-stained area.

### In vivo MRI to evaluate the degree of renal fibrosis

Mice underwent MRI on the 13th day after SK administration with a 7 T small animal MRI system (Agilent 7 T MRI system) and body coil. Anesthesia was induced with 2% isoflurane and maintained with 1.5% isoflurane. The mice were placed in the prone position with their abdomens centered relative to the center of the radiofrequency coil and connected to a respirator sensor to monitor breathing. A RAPID Rat Head System was used for radiofrequency excitation and signal detection. All scans were performed under free breathing. Images were acquired using diffusion tensor imaging (DTI) sequences, and the parameters were as follows: directions = 30 (b = 1004.7 s/mm^2^) + null (b = 0 s/mm^2^), Δ = 4 ms, and δ = 16 ms. The image acquisition parameters were as follows: TR/TE = 3000 ms/50.73 ms, α = 60°, matrix = 128 × 128, FOV = 2 × 2 cm, and imaging slice thickness = 1 mm. The total duration for the diffusion preparation was 4 ms. A scan delay of 16 ms was applied between scans, and two b = 0 s/mm^2^ scans were incorporated to limit the effects of T1 relaxation. The total scan time was approximately 1 h. Due to hydronephrosis renal structural abnormalities caused by ureteral obstruction, only the outer cortex and outer medulla of rat kidney tissue were evaluated for imaging analysis. Fractional anisotropy (FA) maps were obtained and calculated using VNMRJ4.0 software. To accurately measure the MRI signal intensity and determine the degree of fibrosis between different groups, five scanning planes were selected for each kidney sample, and three small regions of interest were randomly selected for each scanning plane.

### Western blot analysis

Total protein was extracted from cells using 500 μL of RIPA protein lysis buffer (Solarbio, Beijing, China). After centrifugation at 10000 (× g) for 10 min, a Bradford protein assay kit (Applygen, Beijing, China) was used for protein quantification. Equivalent amounts of protein (40 μg) were denatured at 100 °C for 10 min in loading buffer (Applygen, Beijing, China), separated by electrophoresis on 8–10% SDS-PAGE gels according to relative molecular weight, and electrotransferred onto 0.45-μm polyvinylidene difluoride membranes (Millipore, Bedford, MA, USA). Then, the membranes were blocked in blocking buffer (Nacalai Tesque, Japan) at 25 ± 5 °C and incubated with primary antibodies, including β-actin (Proteintech Group, USA, 1:5000), α-SMA (Proteintech Group, USA, 1:1000), collagen III (col I) (Abcam, UK, 1:5000), STAT3 (Proteintech Group, USA, 1:2000), p-STAT3 (Tyr705) (Cell Signaling Technology, USA, 1:2000), JAK2 (Cell Signaling Technology, USA, 1:1000), p-JAK2 (Cell Signaling Technology, USA, Tyr1007) (1:1000), and Peroxiredoxin 5 (Prdx5) (Proteintech Group, USA, 1:800), at 4 °C overnight. After being washed in TBST, the membranes were incubated with secondary antibody (Cell Signaling Technology, USA, 1:10000) for 1 h at room temperature and then washed again with TBST. Subsequently, ECL detection kits were used for exposure. Then, the optical density of the protein bands was read and analyzed by Image Lab™ software for quantitative analysis. The densitometric values were normalized to the density of the β-actin band.

### Quantitative real-time PCR

Total RNA was extracted from cells or renal tissue using the Monarch® Total RNA Miniprep Kit according to the manufacturer’s instructions. Then, the GoScript Reverse Transcription System was used to synthesize first-strand cDNA according to the manual. Real-time quantitative reverse transcription PCR was performed in a 20-μL reaction. Gene-targeting primers were designed based on mRNA sequences published by NCBI and are listed in Table 1. Relative mRNA abundance was determined on the basis of the ratio of specific mRNA expression to β-actin mRNA expression in the same sample, and the fold change relative to that in the mice in the sham group or the cells in the 0 ng/mL TGF-β1 group was calculated by the 2^-∆∆Ct^ method.

**Table 1:** Primer pairs used for real-time PCR analysis of target genes

Gene name	Forward primer (5′-3′)	Reverse primer (5′-3′)
β-actin (rat)	CAGCTGAGAGGGAAATCGTG	CGTTGCCAATAGTGATGACC
α-SMA (rat)	CTACATGCGTCTGGACTTGG	CCAGGGAAGAAGAGGAAGCA
JAK2 (rat)	ATGTCAACCAACGTCCCTCT	GAGCTTTGCTCTGGTTCTGG
STAT3 (rat)	GCTGGAACGGCATCTTCAGG	CTGTCTGGTCACAGACTGGT
β-actin (mouse)	TATAAAACCCGGCGGCGCA	TCATCCATGGCGAACTGGTG
α-SMA (mouse)	GTCCCTCTATGCCTCTGGAC	AAGGAATAGCCACGCTCAGT
FSP-1 (mouse)	AGCTGCATTCCAGAAGGTGA	ATGCAGGACAGGAAGACACA
Col I (mouse)	TGGAAACCCGAGGTATGCTT	CATTGCATTGCACGTCATCG
Col III (mouse)	ACTGGTGAACGTGGCTCTAA	AACCTGGAGGACCTGGATTG
FN (mouse)	TCACCTACGGAGAGACAGGA	TGTTGTTGATGGTGGCTGTG
STAT3 (mouse)	TGGTGTCTCCACTTGTCTACCTCTAC	GTGTCACACAGATGAACTTGGTCTT
JAK2 (mouse)	GGAATGGCCTGCCTTACAATG	TGGCTCTATCTGCTTCACAGAAT
TGF-β (mouse)	TTGCTTCAGCTCCACAGAGA	CAGAAGTTGGCATGGTAGCC
SOCS1 (mouse)	CCCTCTTAACCCGGTACTCC	CTCCCACGTGGTTCCAGAAA
SOCS3 (mouse)	GCAGGAGAGCGGATTCTACT	TGGATGCGTAGGTTCTTGGT

### Statistical analysis

All data from at least three independent experiments are expressed as the mean ± standard error of the mean (SEM). Statistical analysis was performed using the SPSS 20.0 software package. Normality tests and homogeneity tests for variance were performed. If the data were normally distributed, Student’s t-test was used to compare the differences between the two groups, and one-way analysis of variance (ANOVA) (LSD test for even variances and Dunnett’s test for uneven variances) was used to compare the differences between groups. If the data were not normally distributed, the nonparametric Mann-Whitney U test was used to compare the differences between two groups, and the nonparametric Kruskal-Wallis test was used to compare the differences between more than two groups. *P* < 0.05, *P* < 0.01 or *P* < 0.001 indicates a significant difference.

## Results

### Effect of SK on NRK-49F cell viability after TGF-β1 treatment

**Fig. 1:**
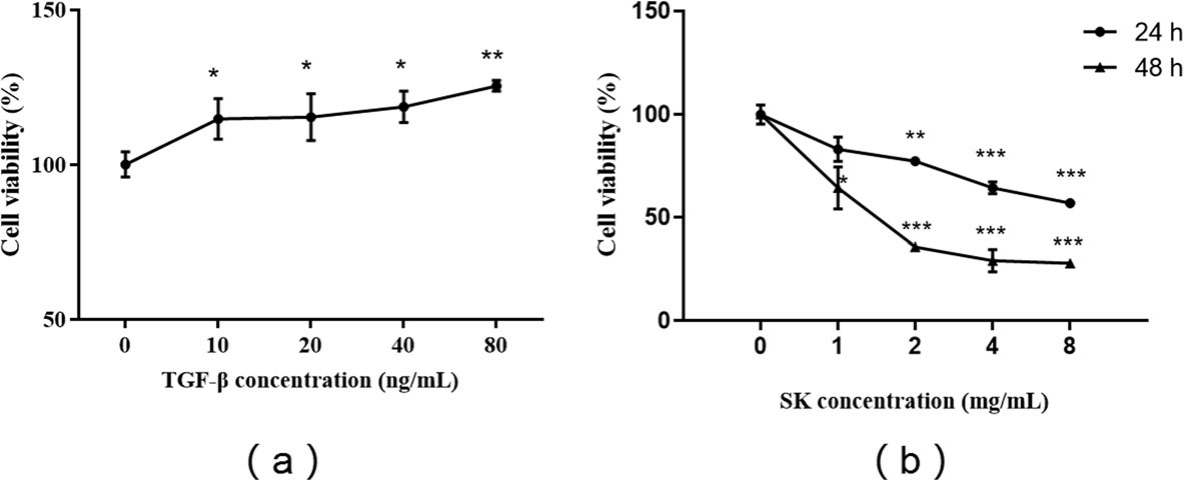
Effect of SK on NRK-49F cell viability after TGF-β1 treatment. The data are presented as the mean ± SEM. **a** Effect of different doses of TGF-β1 on cell viability calculated as a percentage of the viability of control cells after treatment for 24 h; ^*^P < 0.05, ^**^P < 0.01 versus 0 ng/mL TGF-β1 as a control. **b** Effect of different doses of SK on cell viability in the presence of 10 ng/mL TGF-β1 calculated as a percentage of the viability of control cells after treatment for 24 h and 48 h. ^*^P < 0.05, ^**^P < 0.01, ^***^P < 0.001 versus 0 mg/mL SK as a control

TGF-β is a key fibrogenetic cytokine involved in renal fibrosis. We first used different doses of TGF-β1 (0, 10, 20, 40, and 80 ng/mL) to stimulate the growth of NRK-49F cells. Treatment with TGF-β1 for 24 h increased cell viability in a dose-dependent manner (Fig. 1a), which is consistent with previous findings obtained via the MTT assay [17]. Gradient concentrations of SK (1, 2, 4, and 8 mg/mL) significantly reduced the enhancement of viability induced by 10 ng/mL TGF-β1 (Fig. 1b), and the effect of treatment for 48 h was more obvious than that of treatment for 24 h. This result indicated that SK can suppress TGF-β1-induced renal fibroblast growth and enhancement of viability in vitro.

### SK inhibited TGF-β-induced expression of α-SMA and deposition of ECM components in NRK-49F cells

**Fig. 2:**
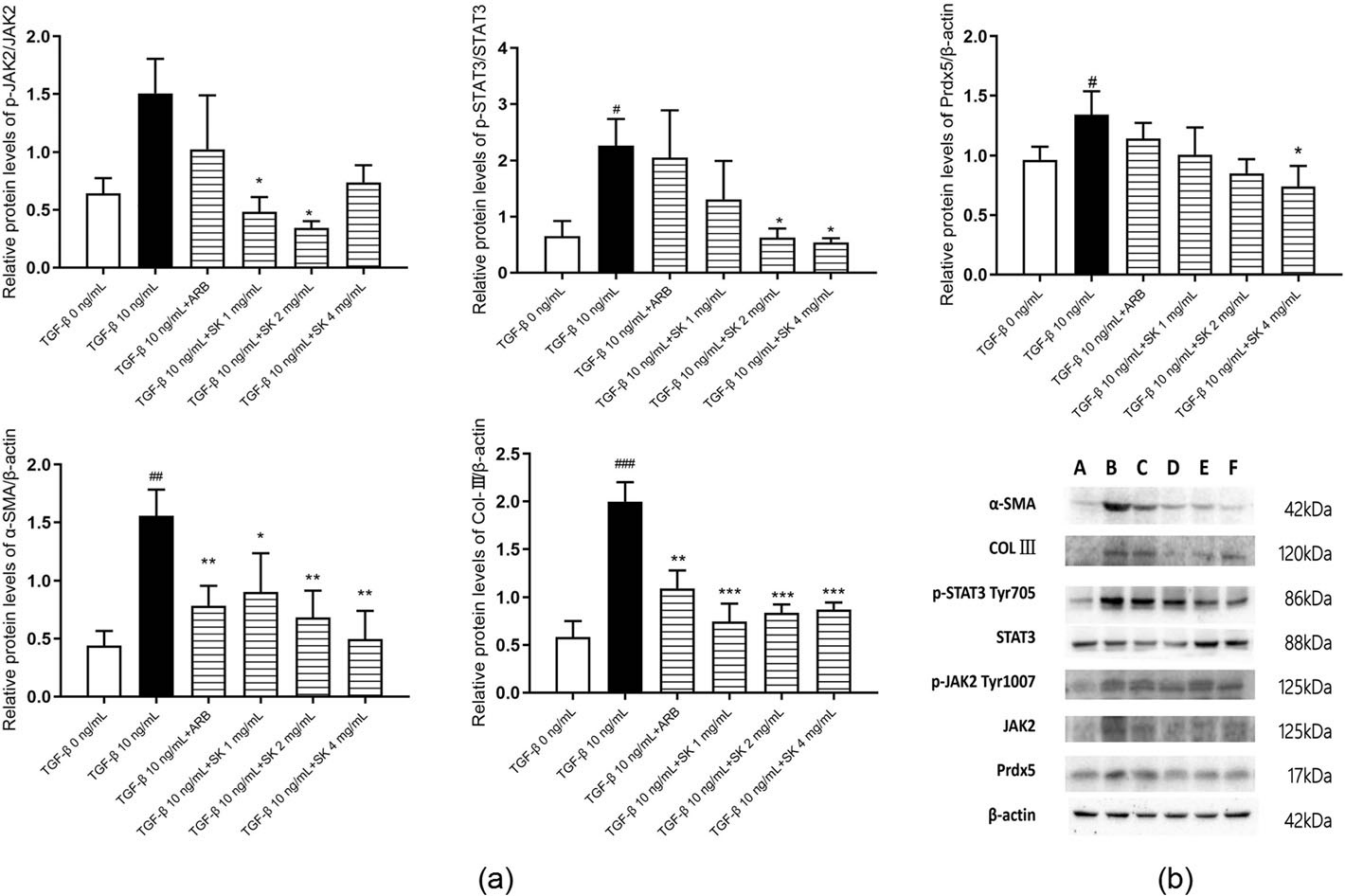
Expression of p-JAK2/JAK2 (Tyr1007), p-STAT3/STAT3 (Tyr705), Prdx5, col. III and α-SMA in each group. The data are expressed as the mean ± SEM (*n* = 3). **a** Expression of each protein relative to that of β-actin in each group; ^#^*P* < 0.05, ^##^*P* < 0.01, ^###^*P* < 0.001 versus 0 ng/mL TGF-β1; *P < 0.05, **P < 0.01, ***P < 0.001 versus 10 ng/mL TGF-β1. **b** Original Western blot results (A = 0 ng/mL TGF-β group, B = 10 ng/mL TGF-β group, C = ARB group, D = 1 mg/mL SK group, E = 2 mg/mL SK group, F = 4 mg/mL SK group)

**Fig. 3:**
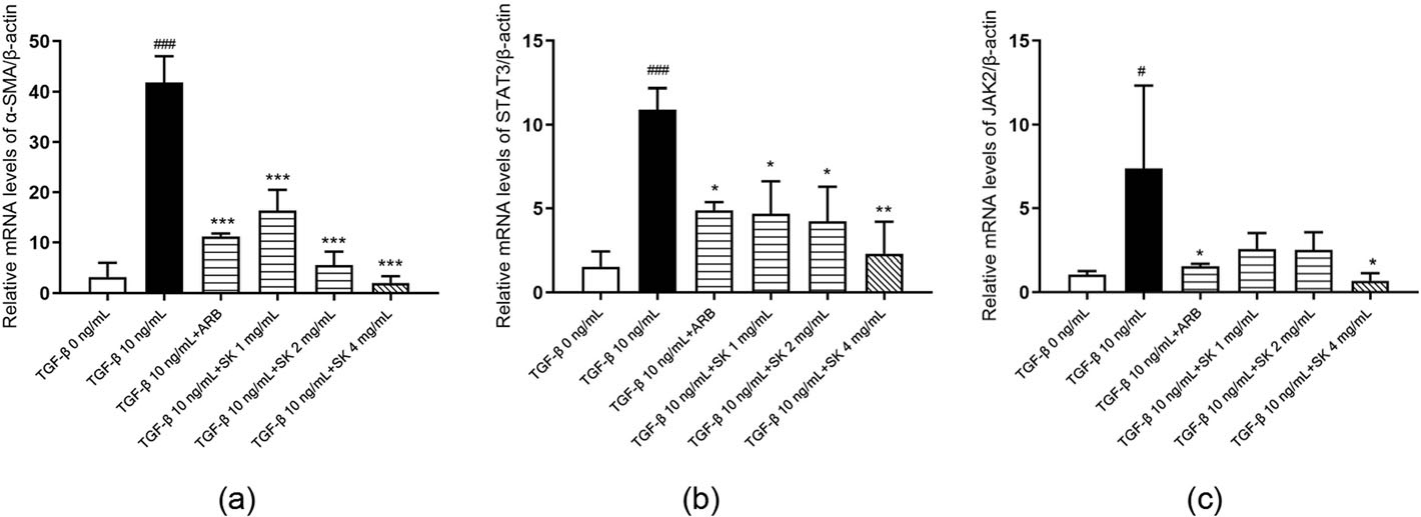
Effects of SK on target gene levels in NRK-49F cells stimulated with TGF-β1. Abundance of **(a)** α-SMA, **(b)** STAT3 and **(c)** JAK2 mRNA relative to that of β-actin in each group of NRK-49F cells. The data are expressed as the mean ± SEM (*n* = 3); ^#^*P* < 0.05, ^##^*P* < 0.01, ^###^*P* < 0.001 versus 0 ng/mL TGF-β1; *P < 0.05, **P < 0.01, ***P < 0.001 versus 10 ng/mL TGF-β1

Myofibroblasts are active fibroblasts characterized by the expression of α-SMA and ECM deposition. TGF-β1 is an important cytokine involved in ECM production by myofibroblasts and fibrosis [18]. As shown in Fig. 2a and b, both α-SMA and col. III were highly expressed in NRK-49F cells after TGF-β1 treatment, indicating that the cultured cells were converted to activated myofibroblasts when administered TGF-β1. To further investigate whether SK could suppress the activation of renal interstitial fibroblasts, we examined the effect of SK on α-SMA and collagen III expression in NRK-49F cells. SK administration markedly decreased the expression of α-SMA (in a dose-dependent manner) (Fig. 2 and 3a) and col. III (Fig. 2), suggesting that SK could directly suppress TGF-β1-induced activation of NRK-49F cells. There was a tendency for the effect of 4 ng/mL SK in decreasing the TGF-β1-induced expression of α-SMA and deposition of ECM components to be superior to that of losartan (1 mg/L).

### SK inhibited TGF-β1-induced STAT3, JAK2 activation and Prdx5 expression in NRK-49F cells

To explore the possible mechanisms by which SK inhibits fibroblast activation, we further detected STAT3 and JAK2 gene expression and protein levels in NRK-49F cells using RT-qPCR and Western blotting, respectively. NRK-49F cells expressed higher levels of STAT3 and JAK2 mRNA after TGF-β1 stimulation than before treatment. SK dramatically inhibited TGF-β1-induced STAT3 and JAK2 mRNA expression at 24 h (Fig. 3b and c). Phosphorylation of STAT3 at Tyr705 and JAK2 at Tyr1007 was increased in the presence of TGF-β1. SK also suppressed the phosphorylation of these two molecules (Fig. 2). Prdx5, a JAK2/STAT3 pathway regulator, was upregulated in the presence of TGF-β1, and this upregulation was also reversed by SK treatment (Fig. 2). Almost all the effects, except for that on the phosphorylation of JAK2 and the expression of col. III, were more robust in the high-dose SK group than in the low-dose SK group. Consequently, it seems clear that SK might inhibit TGF-β1-induced activation of NRK-49F fibroblasts through regulating JAK2/STAT3 activation. At 2 ng/mL or 4 ng/mL, SK had a better effect in ameliorating TGF-β1-induced STAT3 and JAK2 activation and Prdx5 expression than losartan.

### SK alleviated UUO-induced renal dysfunction and renal morphological changes

**Fig. 4:**
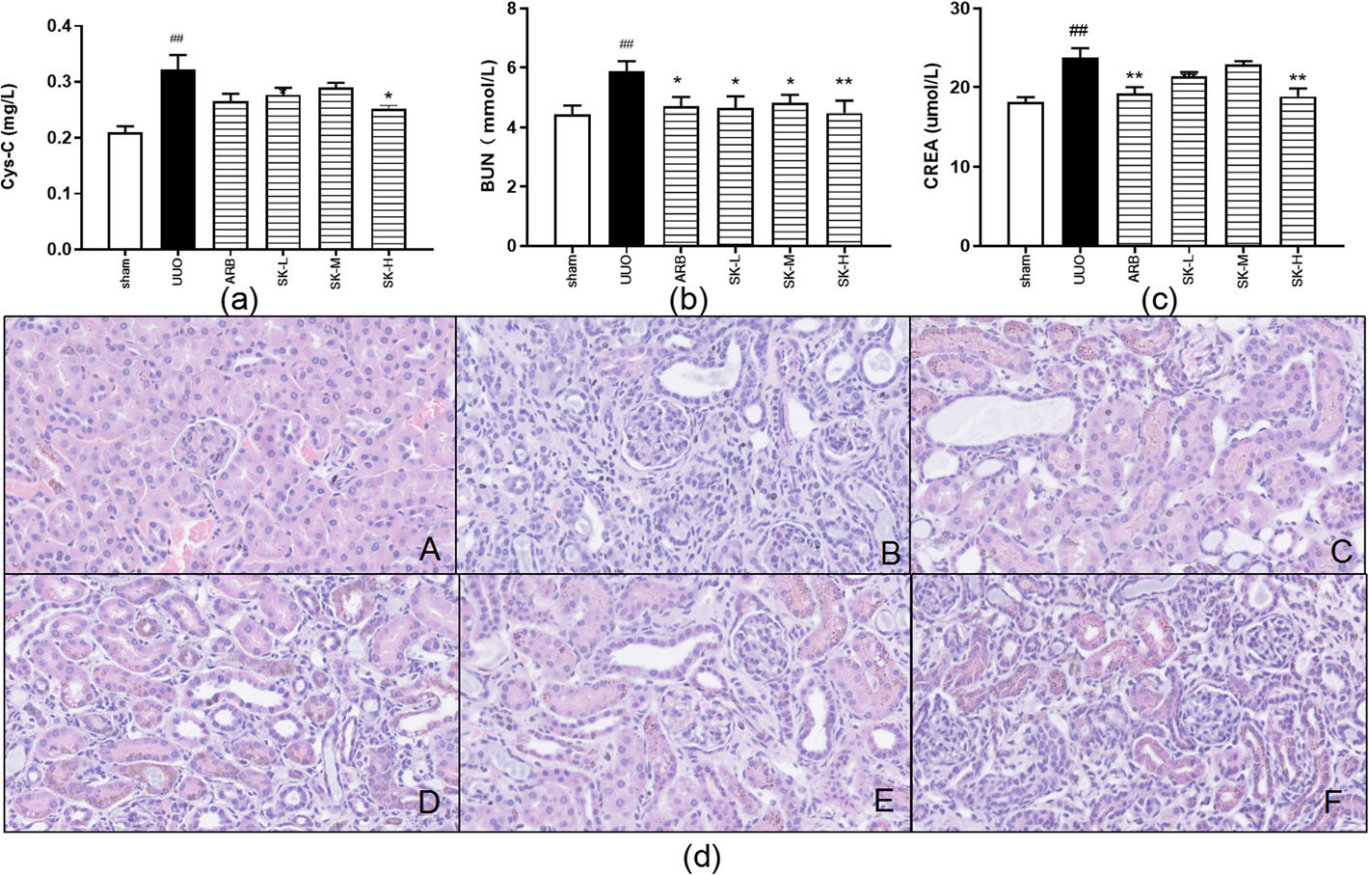
Renal function impairment and pathological lesions were alleviated by SK on the 14th day of treatment. **a** Levels of Cys-C (*n* = 6); **b** levels of BUN (*n* = 6); **c** levels of SCr (*n* = 6); **d** representative images of histopathological manifestations in obstructed kidneys detected on the 14th day (HE staining, 200×). The data are presented as the mean ± SEM; ^#^*P* < 0.05, ^##^*P* < 0.01, ^###^*P* < 0.001 versus the sham group; *P < 0.05, **P < 0.01, ***P < 0.001 versus the UUO group (A = sham group, B = UUO group, C = ARB group, D = low-dose SK group, E = moderate-dose SK group, F = high-dose SK group)

A renal fibrosis mouse model was established by inducing UUO. Figure 4 a, b, c shows the renal dysfunction indexes of the sham group, the UUO group, the ARB group, and the high-, moderate-, and low-dose SK groups after 14 days of treatment. The high dose of SK significantly reduced the Cys-C level (*P* ≤ 0.05; Fig. 4a) and SCr levels (*P* ≤ 0.01; Fig. 4c), and SK markedly decreased the BUN level at all doses (*P* ≤ 0.05 or *P* ≤ 0.01; Fig. 4b). The above results suggested that SK ameliorated renal function impairment and renal fibrosis and that the effects of SK were similar to those of losartan.

Renal HE staining revealed that accumulation of ECM, infiltration of inflammatory cells, dilation and/or atrophy of renal tubules, degeneration and necrosis of renal tubule epithelial cells without obvious changes in glomerular structure in the UUO group (Fig. 4d). Renal function impairment was alleviated to varying degrees in the different treatment groups compared with the UUO group; the moderate dose and high dose of SK achieved the best effects, although the degree of renal function in the sham operation group was not achieved in any of the treatment groups.

### SK decreased the fibrosis-related FA value detected using DTI

**Fig. 5:**
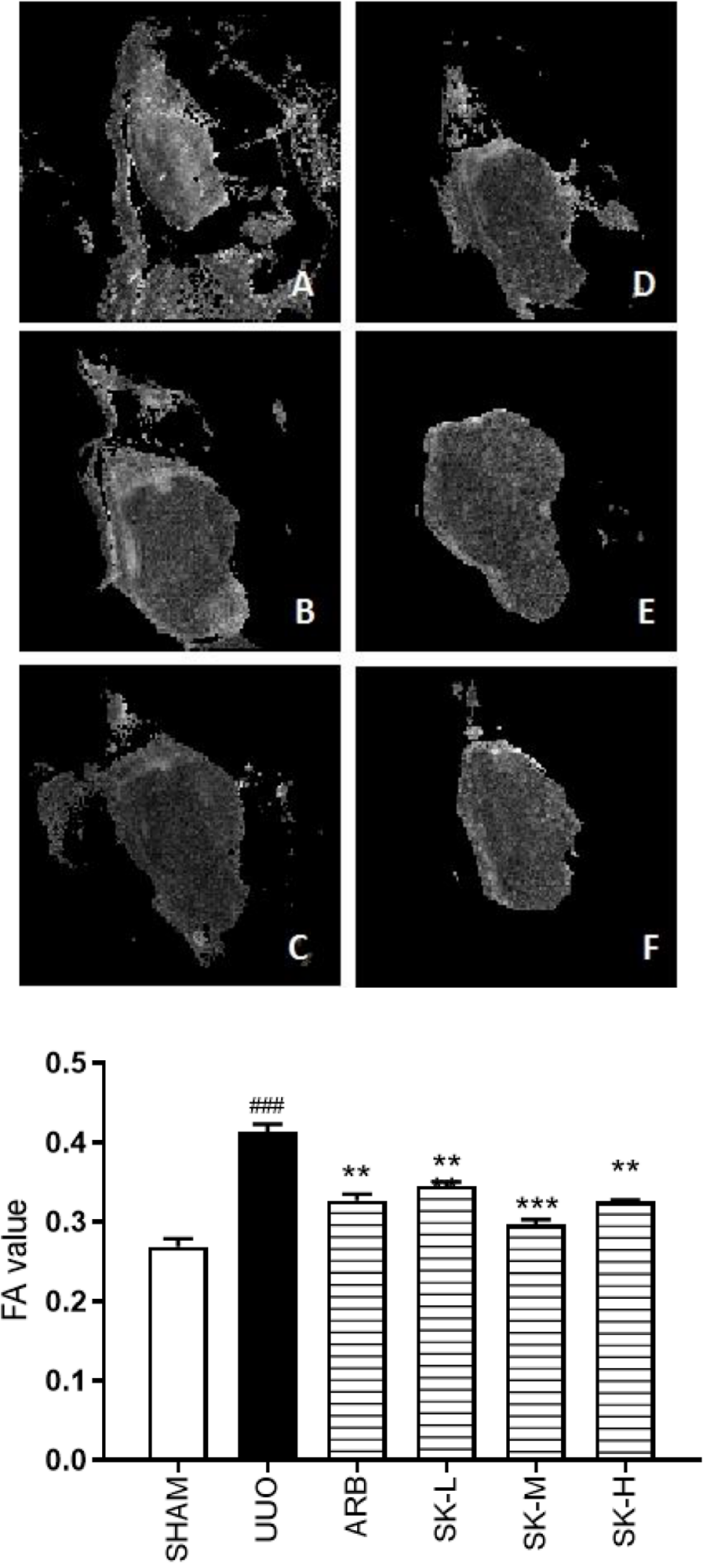
FA map and the quantified signals of FA map were reversed by SK treatment on the 14th day of treatment. The data are presented as the mean ± SEM (*n* = 3); ^#^*P* < 0.05, ^##^*P* < 0.01, ^###^*P* < 0.001 versus the sham group; *P < 0.05, **P < 0.01, ***P < 0.001 versus the UUO group (**A** = sham group, **B** = UUO group, **C** = ARB group, **D** = low-dose SK group, **E** = moderate-dose SK group, **F** = high-dose SK group)

As illustrated by Fig. 5, the FA value which has a linear relationship with the degree of fibrosis in real tissue was determined by in vivo DTI. Compared with that in the sham group, the FA value in the UUO group was significantly increased (*P* < 0.01). The increase in in the FA value in the presence of ureteral obstruction conditions was significantly reversed in the ARB and high-, moderate-, and low-dose SK groups compared to the UUO group (*P* < 0.01 or *P* < 0.001), with moderate-dose SK having the most significant effect (*P* < 0.001).

### SK inhibited fibroblast activation and deposition of ECM components in UUO mice

**Fig. 6:**
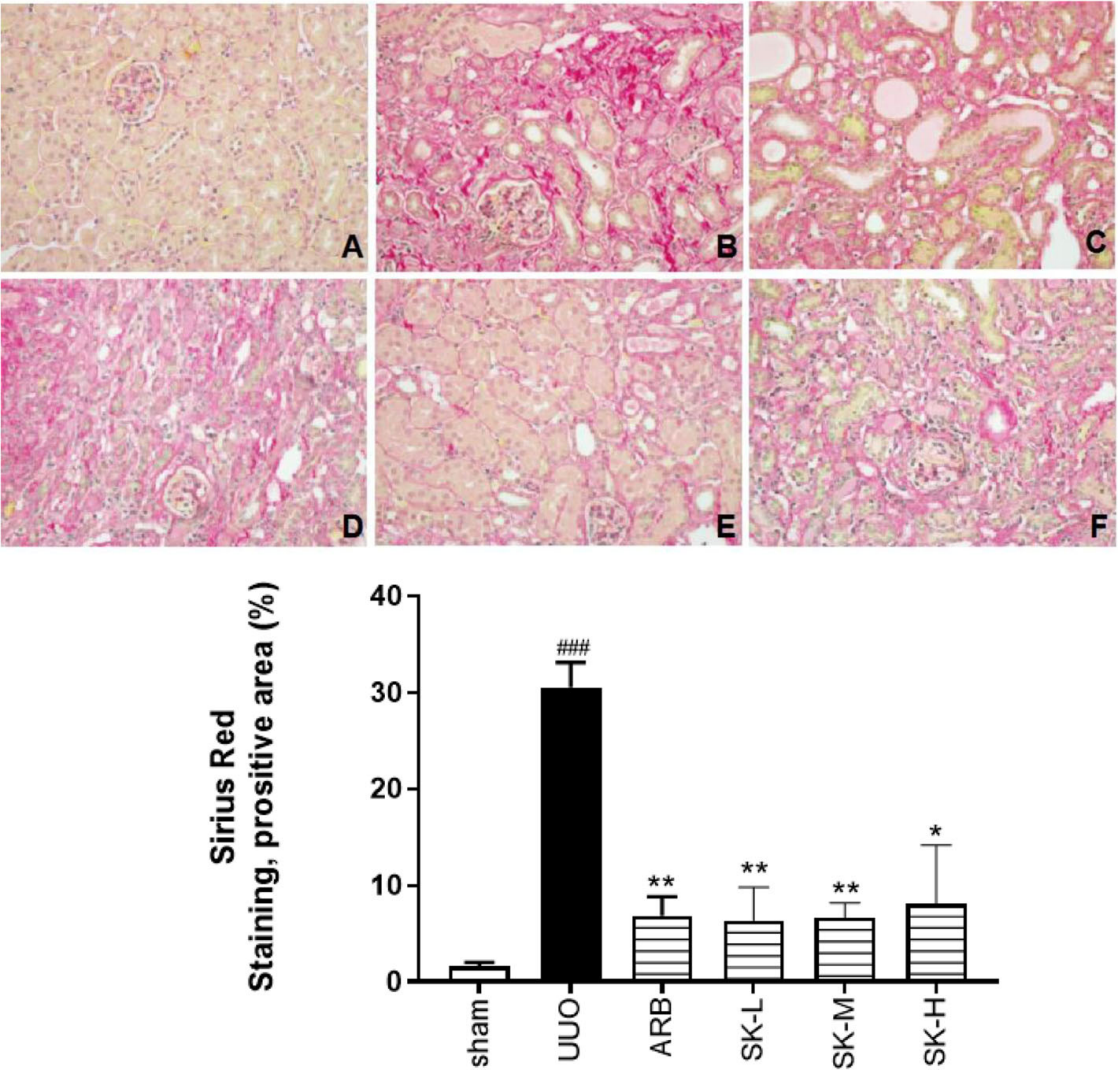
The red area of fibrosis in the affected kidney was reduced by SK on the 14th day of treatment (Sirius red staining, 200 ×). The data are presented as the mean ± SEM (*n* = 3); ^#^*P* < 0.05, ^##^*P* < 0.01, ^###^*P* < 0.001 versus the sham group; *P < 0.05, **P < 0.01, ***P < 0.001 versus UUO group (**A** = sham group, **B** = UUO group, **C** = ARB group, **D** = low-dose SK group, **E** = moderate-dose SK group, **F** = high-dose SK group)

**Fig. 7:**
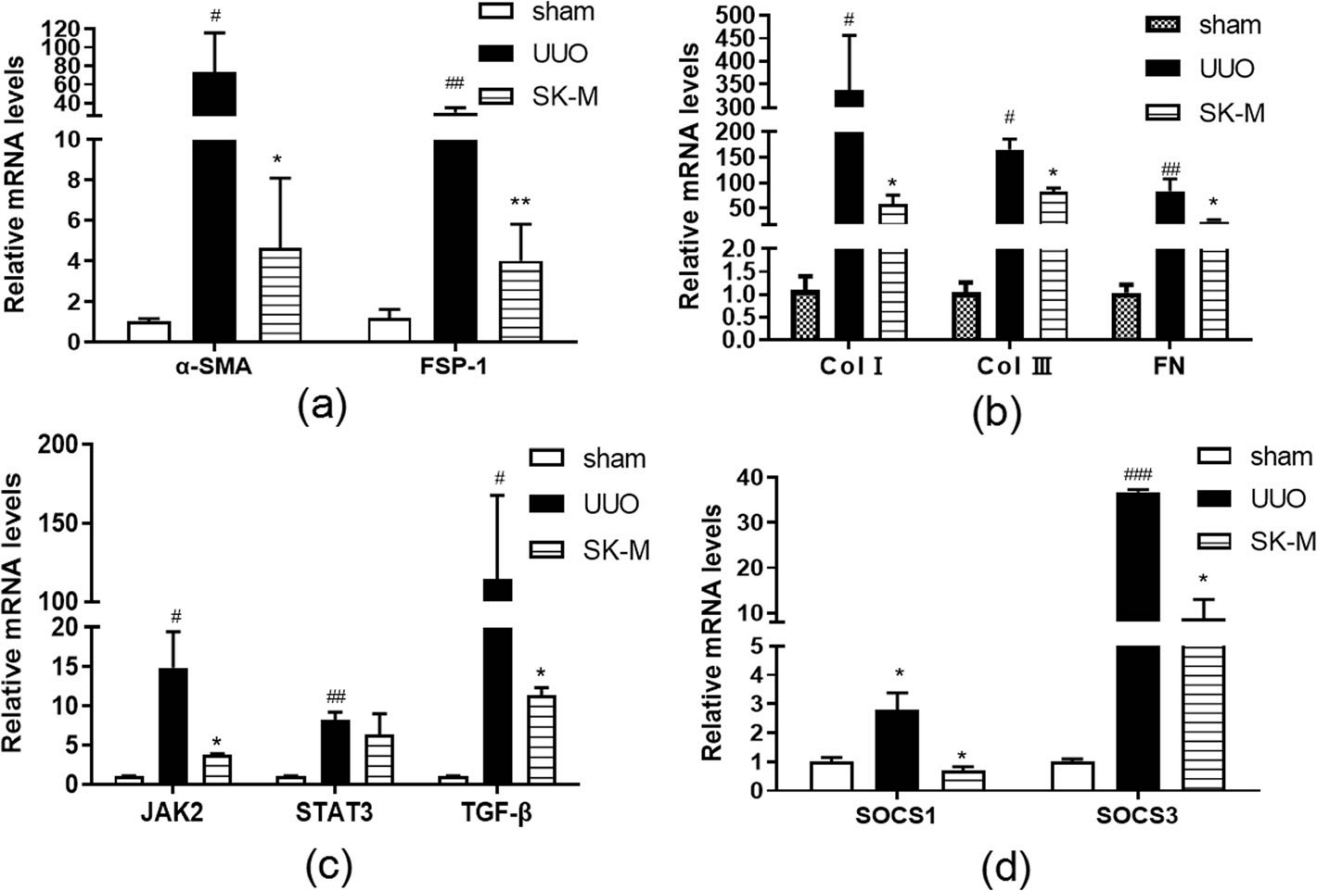
Effects of SK on target gene levels in the obstructed kidneys of UUO mice. mRNA levels of (a) α-SMA and FSP-1; (b) col. I, col. III and FN; (c) JAK2, STAT3, and TGF-β; and (d) SOCS1 and SOCS3 to the β-actin mRNA levels in obstructed kidneys in each group on the 14th day after obstruction. The data are expressed as the mean ± SEM (*n* = 3); ^#^*P* < 0.05, ^##^*P* < 0.01, ^###^*P* < 0.001 versus the sham group; *P < 0.05, **P < 0.01, ***P < 0.001 versus the UUO group

Additionally, we evaluated the expression of the fibroblast activation marker α-SMA, fibroblast-specific protein-1 (FSP-1), and the classical ECM components fibronectin (FN), col. III and collagen I (col I) in the sham, UUO and moderate-dose SK groups using real-time PCR. Because the moderate dose of SK is equal to the clinically recommended dose and because the overall improvement in the moderate-dose group was superior to that in the high and low-dose groups, moderate dose of SK was used for assessing mRNA expression. As shown in Fig. 7a and b, moderate-dose SK significantly alleviated the increases in α-SMA, FSP, FN, col. III and col. I levels. These results were consistent with the Sirius red staining results, which showed that the red area of fibrosis in the affected kidney in the ARB group and the SK moderate- and low-dose groups was significantly smaller than that in the untreated group (*P* < 0.05) (Fig. 6), indicating that SK decreased the number of collagen bundles in the affected kidney.

### Effect of SK on STAT3, JAK2 and TGF-β mRNA levels and the expression of regulatory molecules and the suppressor of cytokine signaling (SOCS) proteins SOCS1 and SOCS3 in UUO mice

As shown in Fig. 7c, the mRNA levels of TGF-β, JAK2 and STAT3 were significantly elevated in the UUO group compared with the sham group on the 14th day. Compared with sham mice, moderate-dose SK-treated UUO mice showed a marked decline in JAK2 and TGF-β mRNA levels on the 14th day after obstruction, but SK did not significantly decrease STAT3 mRNA levels after UUO. This result suggested that the effect of SK on renal fibrosis and fibroblast activation in UUO mice might occur through regulation of JAK2 and STAT3 activation without changes in total STAT3 mRNA expression. The level of TGF-β, the downstream molecule of STAT3, was also decreased by SK in the context of ureteral ligation.

SOCS proteins are considered vital for the negative regulation of JAK/STAT signaling [19]. In response to UUO, SOCS1 and SOCS3 levels increased significantly and were decreased significantly by in the SK group compared to the model group (Fig. 7d). This result suggests that SK could suppress the JAK/STAT pathway to decrease the expression of its negative regulatory molecules as feedback.

## Discussion

Renal tubulointerstitial fibrosis is the ultimate common pathway of end-stage renal disease. Myofibroblasts, which are derived from a variety of cells, including epithelial cells, endothelial cells, and pericytes, through epithelial-mesenchymal transdifferentiation (EMT) or endothelial-mesenchymal transdifferentiation (EndoMT), can help repair damaged tissue by producing ECM and α-SMA to generate contractile tension [20]. Among these different cell types, activated interstitial fibroblasts are the main sources of myofibroblasts, accounting for 50% of these cells [21]. In addition to α-SMA, activated fibroblasts also specifically express FSP-1. TGF-β is defined as a major driver of renal fibrosis and has been found to be activated in various kidney disease models and renal cells. Activated TGF-β binds to the TGF-β receptor and induces downstream signaling molecules, including molecules and transcription factors related to EMT, EndoMT, fibroblast activation, ECM production and inhibition of ECM degradation, through Smad-dependent or Smad-independent pathways to promote protein synthesis or gene expression, thus further enhancing fibrosis and inflammation [22]. Among the downstream pathways of TGF-β, the activation of STAT3 has been found to play a role in renal fibrosis [11]. In response to numerous growth factors and cytokines, including TGF-β [23], STAT3 is activated by Tyr phosphorylation at Tyr705 through Janus kinases. The activated STAT3 protein enters the nucleus in the form of a dimer and binds to the target gene. Moreover, STAT3 induces TGF-β pathway signal transduction [24]. Indeed, in the UUO model, the activation of STAT3 at Tyr705 in renal interstitial fibroblasts, along with histopathological lesions and fibroblast activation, is increased from the first day, peaking at 7 days and rising until 14 days [25]. STAT3 is likely involved in many kinds of kidney diseases through regulation of the expression of target genes, especially the transcription factors involved in the development of CKD. In the present study, on the 14th day after obstruction, the mRNA levels of α-SMA, JAK2 and STAT3 were significantly increased with increased renal fibrosis degree.

According to previous research, SK and its components have been found to reduce pathological damage, inhibit endothelial cell proliferation, alleviate proteinuria and glomerulosclerosis, protect residual renal function and slow disease progression, thus intervening in CKD and renal fibrosis progression; however, there have been no reports on fibroblast activation. According to previous studies and our CCK-8 results, 10 ng/mL TGF-β1 was used to activate fibroblasts; 1, 2, and 4 mg/mL were selected as the low-, moderate- and high-doses of SK; and 48 h was selected as the intervention time in the present study. Upregulation of α-SMA and morphological changes accompanied by cell proliferation and activation, which manifested as increases in cell viability and ECM production, occurred in NRK-49F cells after TGF-β1 stimulation [26, 27]. In this study, it was confirmed that TGF-β1 regulated the gene expression of α-SMA and ECM by activating the upstream JAK2/STAT3 pathway. Our data showed that SK directly inhibited TGF-β1-induced NRK-49F cell activation and ECM production in vitro. Furthermore, we demonstrated that SK could attenuate renal fibrosis in UUO mice in vivo, reducing interstitial ECM accumulation on the 14th day after obstruction. These results indicated that SK could markedly attenuate renal fibrosis by inhibiting interstitial fibroblast activation and reducing α-SMA expression. The potential mechanism of the antifibrotic effect of SK is inhibition of fibroblast activation through regulation of the JAK2/STAT3 signaling pathway both in vitro and in vivo. Moderate-dose SK also significantly reversed the upregulation of TGF-β1 induced by UUO, suggesting that the effect of SK is related to TGF-β. Because TGF-β and STAT3 interact with each other, SK may inhibit STAT3 by targeting TGF-β, downregulate TGF-β by targeting STAT3, or inhibit both. Our previous system pharmacology study predicted STAT3 as one of the possible target molecules of SK [28], and this finding was verified in the present study. We use losartan, an ARB can significantly attenuate renal fibrosis and renal tubular cell apoptosis by inhibiting phosphorylation of the signaling protein STAT3 in a rat model of UUO [29], as a positive control drug. The previous findings are consistent with our data.

Peroxiredoxins are a family of mercaptan-dependent peroxidases that can reduce oxidative stress by catalyzing the reduction of hydrogen peroxide. The level of Prdx5 in the rat kidney decreased on the first day after UUO. It has been observed that Prdx5 expression gradually increases until 5 days after TGF-β treatment. Overexpression of Prdx5 was shown to inhibit the TGF-β-induced phosphorylation of STAT3 and decrease TGF-β-induced α-SMA and FN expression in NRK-49F cells, indicating that Prdx5 negatively regulates TGF-β-induced STAT3 activation and fibrosis in NRK-49F cells [12]. The main regulatory mechanism underlying endogenous STAT3 signaling involves the SOCS protein family. After STAT3 is activated by cytokines, the STAT3 dimer is transported to downstream target genes of nuclear induction, including SOCS [30]. Specifically, the kinase-inhibitory region of SOCS1 can directly bind to JAK2 and inhibit STAT3 phosphorylation [31]. UUO-induced JAK/STAT activation causes SOCS expression elevation [32]. After stimulation with 10 ng/mL TGF-β1 for 48 h, Prdx5 protein expression in NRK-49F cells increased significantly, which is consistent with previous results. SK (4 mg/ml) significantly reduced Prdx5 protein levels after 48 h of intervention. On the 14th day after UUO, the levels of SOCS1 and SOCS3 mRNA in the affected kidney were significantly upregulated in the UUO group compared with the control group, indicating that the activation of STAT3 signaling after UUO induced the expression of SOCS1 and SOCS3 to negatively regulate the overactivated STAT3 signal. After 14 days of SK intervention, the levels of SOCS1 and SOCS3 mRNA decreased significantly. These results showed that instead of directly elevating negative regulators to inhibit JAK2/STAT3 signaling, SK inhibited JAK2/STAT3, resulting in downregulation of the negative regulators Prdx5, SOCS1 and SOCS3. Thus, JAK2/STAT3 could be a direct therapeutic target of SK for renal fibrosis.

MRI is a very safe method for assessing structural and physiological changes in the kidney without the use of an intravenous contrast agent [33, 34]. Diffusion-weighted imaging (DWI) is a well-known MRI method that is used to image molecular movement or diffusion reflecting changes in the microstructure of biological tissues [35]. DTI is a new imaging technology developed based on diffusion-weighted imaging (DWI) that can describe not only the speed of water molecules but also the direction of their movement, namely, anisotropy. Compared with DWI, DTI is more sensitive and accurate in reflecting the change in water dispersion. DTI can also provide additional information about the direction and amount of diffusion measured on the basis of FA. Hueper et al. [36] showed that in DN rats, FA was related to glomerulosclerosis, interstitial fibrosis and renal tubular injury. Yan et al. [37] found that FA may be a biomarker of early DN. Kaimori et al. [38] observed a linear relationship between the FA value and the degree of real tissue fibrosis in a UUO rat model. In this study, we optimized the parameters to reduce the scanning time, anesthesia time and consumption of experimental resources. The DTI results were consistent with Sirius red staining in terms of the degree of renal fibrosis caused by UUO and the ability of SK to alleviate renal fibrosis. To some extent, these findings revealed that SK has a real effect on renal fibrosis in vivo. In this study, we demonstrated the mechanism by which SK regulates renal interstitial fibrosis both in vivo and in vitro. In future studies, the use of inhibitors or siRNAs to inhibit JAK2/STAT3 is required to further verify this mechanism.

## Conclusions

In conclusion, our data suggest that SK may effectively inhibit renal fibroblast activation and renal fibrosis in UUO mice by regulating the JAK2/STAT3 signaling pathway. Our results provide a good understanding of the mechanism of SK in treating renal disease. However, the detailed molecular mechanisms of SK treatment in renal fibrosis require further exploration.

## Supplementary Information

**Additional file 1: Table S1.** Cell viability (%) calculated as a percentage of control cell viability in each group after treatment for 24 h. **Table S2.** Cell viability (%) calculated as a percentage of control cell viability in each group after SK treatment. **Table S3.** Expression of p-JAK2/JAK2 (Try1007), p-STAT3/STAT3 (Try705), Prdx5, col. III and α-smooth muscle actin (α-SMA) in each group of NRK-49F. **Table S4.** Abundance of α-SMA, JAK2, STAT3 mRNA of NRK-49F relative to β-actin in each group. **Table S5.** Levels of Scr, BUN and CYS-C in each group of mice on the 14th day of obstruction. **Table S6.** Quantification of signals of FA map in each group of mice on the 13th day of obstruction. **Table S7.** Red positive fibrosis areas of sirius red staining of affected kidney in each group of mice on the 14th day of obstruction. **Table S8.** Abundance of α-SMA, FSP-1, col. I, col. III, FN, JAK2, STAT3, TGF-β, SOCS1, SOCS3 mRNA relative to β-actin in obstructed kidneys in each group on the 14th day after obstruction.

**Additional file 2: Figure S1.** Expression of α-SMA in each group (A = 0 ng/mL TGF-β group, B = 10 ng/mL TGF-β group, C = ARB group, D = 1 mg/mL SK group, E = 2 mg/mL SK group, F = 4 mg/mL SK group). **Figure S2.** Expression of beta-actin in each group (A = 0 ng/mL TGF-β group, B = 10 ng/mL TGF-β group, C = ARB group, D = 1 mg/mL SK group, E = 2 mg/mL SK group, F = 4 mg/mL SK group). **Figure S3.** Expression of col. III in each group (A = 0 ng/mL TGF-β group, B = 10 ng/mL TGF-β group, C = ARB group, D = 1 mg/mL SK group, E = 2 mg/mL SK group, F = 4 mg/mL SK group). **Figure S4.** Expression of JAK2 in each group (A = 0 ng/mL TGF-β group, B = 10 ng/mL TGF-β group, C = ARB group, D = 1 mg/mL SK group, E = 2 mg/mL SK group, F = 4 mg/mL SK group). **Figure S5.** Expression of p-JAK2 in each group (A = 0 ng/mL TGF-β group, B = 10 ng/mL TGF-β group, C = ARB group, D = 1 mg/mL SK group, E = 2 mg/mL SK group, F = 4 mg/mL SK group). **Figure S6.** Expression of Prdx5 in each group (A = 0 ng/mL TGF-β group, B = 10 ng/mL TGF-β group, C = ARB group, D = 1 mg/mL SK group, E = 2 mg/mL SK group, F = 4 mg/mL SK group). **Figure S7.** Expression of p-STAT3 in each group (A = 0 ng/mL TGF-β group, B = 10 ng/mL TGF-β group, C = ARB group, D = 1 mg/mL SK group, E = 2 mg/mL SK group, F = 4 mg/mL SK group). **Figure S8.** Expression of STAT3 in each group (A = 0 ng/mL TGF-β group, B = 10 ng/mL TGF-β group, C = ARB group, D = 1 mg/mL SK group, E = 2 mg/mL SK group, F = 4 mg/mL SK group).

## Data Availability

The more detailed data used to support the findings of this study are available in the supplementary information.

## Abbreviations

ARB: Angiotensin receptor blocker
BUN: Blood urea nitrogen
CKD: Chronic kidney disease
col. I: Collagen I
col. III: Collagen III
Cys-C: Cystatin C
DTI: Diffusion tensor imaging
DWI: Diffusion-weighted imaging
EndoMT: Endothelial-mesenchymal transdifferentiation
EMT: Epithelial-mesenchymal transdifferentiation
ECM: Extracellular matrix
FSP-1: Fibroblast-specific protein-1
FN: Fibronectin
FA: Fractional anisotropy
JAK/STAT: Janus kinase/signal transducer and activator of transcription
Prdx5: Peroxiredoxin 5
SCr: Serum creatinine
SK: Shenkang
SOCS: Suppressor of cytokine signaling
TGF-β: Transforming growth factor β
UUO: Unilateral ureteral obstruction
α-SMA: α-smooth muscle actin
