# Supplementary material for: Effect of Shenkang on renal fibrosis and activation of renal interstitial fibroblasts through the JAK2/STAT3 pathway
Source: BMC Complement Med Ther. 2021 Jan 6;21:12. doi: 10.1186/s12906-020-03180-3 (PMC7789243; doi:10.1186/s12906-020-03180-3)
Supplement: Supplementary file 1 — Additional file 1: Table S1. Cell viability (%) calculated as a percentage of control cell viability in each group after treatment for 24 h. Table S2. Cell viability (%) calculated as a percentage of control cell viability in each group after SK treatment. Table S3. Expression of p-JAK2/JAK2 (Try1007), p-STAT3/STAT3 (Try705), Prdx5, col. III and α-smooth muscle actin (α-SMA) in each group of NRK-49F. Table S4. Abundance of α-SMA, JAK2, STAT3 mRNA of NRK-49F relative to β-actin in each group. Table S5. Levels of Scr, BUN and CYS-C in each group of mice on the 14th day of obstruction. Table S6. Quantification of signals of FA map in each group of mice on the 13th day of obstruction. Table S7. Red positive fibrosis areas of sirius red staining of affected kidney in each group of mice on the 14th day of obstruction. Table S8. Abundance of α-SMA, FSP-1, col. I, col. III, FN, JAK2, STAT3, TGF-β, SOCS1, SOCS3 mRNA relative to β-actin in obstructed kidneys in each group on the 14th day after obstruction. [file 12906_2020_3180_MOESM1_ESM.docx]

| Group | TGF-β 0 ng/mL | TGF-β 10 ng/mL | TGF-β 20 ng/mL | TGF-β 40 ng/mL | TGF-β 80 ng/mL |
| --- | --- | --- | --- | --- | --- |
|  | 110.388 | 136.698 | 139.1215 | 132.5978 | 125.8744 |
|  | 108.4507 | 106.766 | 102.7143 | 109.9579 | 123.0703 |
|  | 94.78428 | 123.162 | 127.6719 | 128.2767 | 131.007 |
|  | 97.93304 | 103.9466 | 103.117 | 116.9849 | 126.5463 |
|  | 88.44398 | 103.3806 | 104.1949 | 105.881 | 120.9463 |

**Table S1.** Cell viability (%) calculated as a percentage of control cell viability in each group after treatment for 24 h

**Table S2.** Cell viability (%) calculated as a percentage of control cell viability in each group after SK treatment

| Time | 24 h |  |  |  |  |
| --- | --- | --- | --- | --- | --- |
| Group | SK 0 mg/mL | SK 1 mg/mL | SK 2 mg/mL | SK 4 mg/mL | SK 8 mg/mL |
|  | 97.8889 | 103.5373 | 72.7497 | 54.33519 | 53.94227 |
|  | 113.425 | 88.08199 | 75.4565 | 72.43933 | 55.74596 |
|  | 108.1302 | 80.31945 | 83.90381 | 63.68739 | 63.83065 |
|  | 90.515 | 70.32459 | 80.5993 | 66.9502 | 59.01231 |
|  | 90.04086 | 73.36002 | 73.82148 | 63.90047 | 52.04354 |
| Time | 48 h |  |  |  |  |
| Group | SK 0 mg/mL | SK 1 mg/mL | SK 2 mg/mL | SK 4 mg/mL | SK 8 mg/mL |
|  | 102.1034 | 67.93967 | 31.07088 | 19.28024 | 25.32423 |
|  | 97.96473 | 54.7187 | 35.97994 | 28.25852 | 31.59168 |
|  | 104.1047 | 47.64492 | 31.52888 | 49.81756 | 30.12056 |
|  | 98.12951 | 102.3458 | 42.46574 | 21.75697 | 30.13351 |
|  | 97.69772 | 49.15732 | 38.25198 | 26.76783 | 22.724 |

**Table S3.** Expression of p-JAK2/JAK2 (Try1007), p-STAT3/STAT3 (Try705), Prdx5, col Ⅲ and α-smooth muscle actin (α-SMA) in each group of NRK-49F

|  | p-STAT3/STAT3 |  |  |  |  |  |
| --- | --- | --- | --- | --- | --- | --- |
| Group | TGF-β 0ng/mL | TGF-β 10ng/mL | TGF-β +ARB | TGF-β +SK 1mg/ml | TGF-β+SK 2mg/ml | TGF-β+SK 4mg/ml |
|  | 1.19 | 2.99 | 1.83 | 0.76 | 0.84 | 0.51 |
|  | 0.34 | 2.44 | 3.6 | 2.67 | 0.73 | 0.43 |
|  | 0.42 | 1.37 | 0.72 | 0.5 | 0.31 | 0.68 |
|  | Prdx5 |  |  |  |  |  |
| Group | TGF-β 0ng/mL | TGF-β 10ng/mL | TGF-β +ARB | TGF-β +SK 1mg/ml | TGF-β+SK 2mg/ml | TGF-β+SK 4mg/ml |
|  | 0.8486145 | 0.9699689 | 0.9032518 | 0.6460387 | 0.6224753 | 0.4135967 |
|  | 0.8671418 | 1.640154 | 1.35608 | 1.430356 | 0.9171913 | 0.9931965 |
|  | 1.184242 | 1.41213 | 1.17188 | 0.9530064 | 1.01802 | 0.821622 |
|  | α-SMA |  |  |  |  |  |
| Group | TGF-β 0ng/mL | TGF-β 10ng/mL | TGF-β +ARB | TGF-β +SK 1mg/ml | TGF-β+SK 2mg/ml | TGF-β+SK 4mg/ml |
|  | 0.6277007 | 1.95913 | 1.114742 | 1.485036 | 0.6056739 | 0.3379895 |
|  | 0.4975294 | 1.536535 | 0.7030287 | 0.341565 | 0.3294051 | 0.1775194 |
|  | 0.1936879 | 1.176708 | 0.5399844 | 0.8859878 | 1.117767 | 0.976791 |
|  | COL-Ⅲ |  |  |  |  |  |
| Group | TGF-β 0ng/mL | TGF-β 10ng/mL | TGF-β +ARB | TGF-β +SK 1mg/ml | TGF-β+SK 2mg/ml | TGF-β+SK 4mg/ml |
|  | 0.5925493 | 2.188637 | 1.421457 | 1.061064 | 0.7110338 | 0.8958695 |
|  | 0.2934311 | 1.587626 | 1.087914 | 0.7651649 | 1.008169 | 0.9879723 |
|  | 0.8683386 | 2.217615 | 0.7769008 | 0.4059339 | 0.7900842 | 0.7300665 |
|  | p-JAK2/JAK2 |  |  |  |  |  |
| Group | TGF-β 0ng/mL | TGF-β 10ng/mL | TGF-β +ARB | TGF-β +SK 1mg/ml | TGF-β+SK 2mg/ml | TGF-β+SK 4mg/ml |
|  | 0.38 | 0.93 | 0.4 | 0.26 | 0.28 | 0.71 |
|  | 0.8 | 1.66 | 1.93 | 0.7 | 0.29 | 1.01 |
|  | 0.75 | 1.93 | 0.75 | 0.49 | 0.46 | 0.5 |

**Table S4.** Abundance of α-SMA, JAK2, STAT3 mRNA of NRK-49F relative to β-actin in each group

|  | JAK2 |  |  |  |  |  |
| --- | --- | --- | --- | --- | --- | --- |
| Group | TGF-β 0ng/mL | TGF-β 10ng/mL | TGF-β +ARB | TGF-β +SK 1mg/ml | TGF-β+SK 2mg/ml | TGF-β+SK 4mg/ml |
|  | 1.348791 | 2.569819 | 1.812943 | 4.422923 | 4.621411 | 0.4297789 |
|  | 1.171481 | 17.24771 | 1.56013 | 2.006943 | 1.281944 | 1.578258 |
|  | 0.6328783 | 2.33216 | 1.302846 | 1.268685 | 1.630145 | 0.08180503 |
|  | STAT3 |  |  |  |  |  |
| Group | TGF-β 0ng/mL | TGF-β 10ng/mL | TGF-β +ARB | TGF-β +SK 1mg/ml | TGF-β+SK 2mg/ml | TGF-β+SK 4mg/ml |
|  | 3.260289 | 9.328639 | 4.069919 | 8.272557 | 1.855318 | 0.5605831 |
|  | 0.2881716 | 9.952093 | 4.817663 | 4.22319 | 2.499547 | 0.2178892 |
|  | 1.06437 | 13.43881 | 5.782393 | 1.543993 | 8.349366 | 6.105037 |
|  | α-SMA |  |  |  |  |  |
| Group | TGF-β 0ng/mL | TGF-β 10ng/mL | TGF-β +ARB | TGF-β +SK 1mg/ml | TGF-β+SK 2mg/ml | TGF-β+SK 4mg/ml |
|  | 8.893663 | 32.58443 | 10.74886 | 24.07467 | 9.017814 | 0.9677979 |
|  | 0.6474218 | 42.11059 | 12.40438 | 9.868137 | 0.4546341 | 4.733075 |
|  | 0.1736729 | 50.66009 | 10.45493 | 15.20118 | 7.290972 | 0.4783409 |

**Table S5.** Levels of Scr, BUN and CYS-C in each group of mice on the 14^th^ day of obstruction

|  | serum CREA |  |  |  |  |  |
| --- | --- | --- | --- | --- | --- | --- |
| Group | sham | UUO | ARB | SK-H | SK-M | SK-L |
|  | 16.11 | 21.42 | 19.19 | 20.81 | 22.18 | 20.69 |
|  | 18.37 | 25.35 | 19.1 | 17.42 | 22.12 | 22.12 |
|  | 20.11 | 22.61 | 19.77 | 20.81 | 22.27 | 19.8 |
|  | 16.69 | 26.61 | 20.93 | 14.89 | 24.41 | 20.75 |
|  | 18.37 | 26.76 | 20.87 | 21.02 | 22.49 | 21.51 |
|  | 19.19 | 19.92 | 15.68 | 18.18 | 23.92 | 23.55 |
|  | BUN |  |  |  |  |  |
| Group | sham | UUO | ARB | SK-H | SK-M | SK-L |
|  | 4.34 | 6.81 | 5.76 | 5.97 | 5.58 | 3.68 |
|  | 4.7 | 6.33 | 3.62 | 4.79 | 4.88 | 5.49 |
|  | 4.1 | 6.36 | 4.82 | 4.43 | 5.19 | 5.12 |
|  | 5.19 | 6 | 4.28 | 4.82 | 4.79 | 3.47 |
|  | 5.03 | 4.64 | 4.49 | 3.8 | 4.82 | 5.76 |
|  | 3.2 | 5.12 | 5.25 | 3.11 | 3.65 | 4.31 |
|  | CYS-C |  |  |  |  |  |
| Group | sham | UUO | ARB | SK-H | SK-M | SK-L |
|  | 0.21 | 0.4 | 0.3 | 0.26 | 0.27 | 0.27 |
|  | 0.18 | 0.32 | 0.22 | 0.27 | 0.3 | 0.22 |
|  | 0.21 | 0.36 | 0.23 | 0.25 | 0.31 | 0.29 |
|  | 0.18 | 0.25 | 0.29 | 0.26 | 0.26 | 0.28 |
|  | 0.25 | 0.24 | 0.28 | 0.24 | 0.3 | 0.31 |
|  | 0.23 | 0.36 | 0.27 | 0.23 | 0.3 | 0.29 |

**Table S6.** Quantification of signals of FA map in each group of mice on the 13^th^ day of obstruction

| Group | sham | UUO | ARB | SK-H | SK-M | SK-L |
| --- | --- | --- | --- | --- | --- | --- |
|  | 0.2835111 | 0.4130563 | 0.3116947 | 0.3272962 | 0.3086478 | 0.3404731 |
|  | 0.27343 | 0.4312714 | 0.3320308 | 0.3232607 | 0.293836 | 0.3395846 |
|  | 0.2505111 | 0.3980857 | 0.3381632 | 0.3280948 | 0.2883105 | 0.3561238 |

**Table S7.** Red positive fibrosis areas of sirius red staining of affected kidney in each group of mice on the 14^th^ day of obstruction

| Group | sham | UUO | ARB | SK-H | SK-M | SK-L |
| --- | --- | --- | --- | --- | --- | --- |
|  | 1.4148 | 28.0806 | 9.1494 | 15.1168 | 5.7658 | 7.9512 |
|  | 1.1856 | 30.3944 | 5.6664 | 4.548 | 8.4486 | 2.4906 |
|  | 2.0856 | 33.1908 | 5.843 | 4.5978 | 5.852 | 8.7476 |

**Table S8.** Abundance of α-SMA, FSP-1, col Ⅰ, col Ⅲ, FN, JAK2, STAT3, TGF-β, SOCS1, SOCS3 mRNA relative to β-actin in obstructed kidneys in each group on the 14^th^ day after obstruction

|  | α-SMA |  |  |
| --- | --- | --- | --- |
| Group | sham | UUO | SK-M |
|  | 0.991564 | 13.54271 | 1.040861 |
|  | 1.282932 | 154.8196 | 11.53365 |
|  | 0.7860962 | 52.56793 | 1.395818 |
|  | FSP-1 |  |  |
| Group | sham | UUO | SK-M |
|  | 1.72 | 30.08 | 1.84 |
|  | 1.54 | 21.27 | 7.62 |
|  | 0.38 | 39.05 | 2.56 |
|  | JAK2 |  |  |
| Group | sham | UUO | SK-M |
|  | 0.94 | 22.24 | 3.53 |
|  | 1.21 | 15.98 | 4.06 |
|  | 0.88 | 6.41 | 3.71 |
|  | STAT3 |  |  |
| Group | sham | UUO | SK-M |
|  | 1.205344 | 7.697791 | 1.083809 |
|  | 0.8552656 | 10.16904 | 8.250288 |
|  | 0.9700366 | 6.678131 | 9.642749 |
|  | TGF-β |  |  |
| Group | sham | UUO | SK-M |
|  | 1.09 | 217.6 | 10.38 |
|  | 0.82 | 86.96 | 10.35 |
|  | 1.13 | 37.85 | 13.32 |
|  | ColⅠ |  |  |
| Group | sham | UUO | SK-M |
|  | 1.19 | 221.66 | 60.08 |
|  | 1.57 | 575.59 | 87.56 |
|  | 0.53 | 215.1 | 29.46 |
|  | Col Ⅲ |  |  |
| Group | sham | UUO | SK-M |
|  | 1.35 | 142.46 | 93.99 |
|  | 1.17 | 146.81 | 66.62 |
|  | 0.63 | 206.66 | 83.93 |
|  | FN |  |  |
| Group | sham | UUO | SK-M |
|  | 1.17 | 130.84 | 22.92 |
|  | 0.68 | 47.78 | 30 |
|  | 1.26 | 70.85 | 19.43 |
|  | SOCS1 |  |  |
| Group | sham | UUO | SK-M |
|  | 1.27 | 1.61 | 0.49 |
|  | 0.92 | 3.09 | 0.88 |
|  | 0.86 | 3.63 | 0.76 |
|  | SOCS3 |  |  |
| Group | sham | UUO | SK-M |
|  | 1.19 | 37.85 | 0.85 |
|  | 0.9 | 36.43 | 14.41 |
|  | 0.93 | 35.97 | 11.41 |
