# Supplementary material for: Effect of Shenkang on renal fibrosis and activation of renal interstitial fibroblasts through the JAK2/STAT3 pathway
Source: BMC Complement Med Ther. 2021 Jan 6;21:12. doi: 10.1186/s12906-020-03180-3 (PMC7789243; doi:10.1186/s12906-020-03180-3)
Supplement: Supplementary file 2 — Additional file 2: Figure S1. Expression of α-SMA in each group (A = 0 ng/mL TGF-β group, B = 10 ng/mL TGF-β group, C = ARB group, D = 1 mg/mL SK group, E = 2 mg/mL SK group, F = 4 mg/mL SK group). Figure S2. Expression of beta-actin in each group (A = 0 ng/mL TGF-β group, B = 10 ng/mL TGF-β group, C = ARB group, D = 1 mg/mL SK group, E = 2 mg/mL SK group, F = 4 mg/mL SK group). Figure S3. Expression of col. III in each group (A = 0 ng/mL TGF-β group, B = 10 ng/mL TGF-β group, C = ARB group, D = 1 mg/mL SK group, E = 2 mg/mL SK group, F = 4 mg/mL SK group). Figure S4. Expression of JAK2 in each group (A = 0 ng/mL TGF-β group, B = 10 ng/mL TGF-β group, C = ARB group, D = 1 mg/mL SK group, E = 2 mg/mL SK group, F = 4 mg/mL SK group). Figure S5. Expression of p-JAK2 in each group (A = 0 ng/mL TGF-β group, B = 10 ng/mL TGF-β group, C = ARB group, D = 1 mg/mL SK group, E = 2 mg/mL SK group, F = 4 mg/mL SK group). Figure S6. Expression of Prdx5 in each group (A = 0 ng/mL TGF-β group, B = 10 ng/mL TGF-β group, C = ARB group, D = 1 mg/mL SK group, E = 2 mg/mL SK group, F = 4 mg/mL SK group). Figure S7. Expression of p-STAT3 in each group (A = 0 ng/mL TGF-β group, B = 10 ng/mL TGF-β group, C = ARB group, D = 1 mg/mL SK group, E = 2 mg/mL SK group, F = 4 mg/mL SK group). Figure S8. Expression of STAT3 in each group (A = 0 ng/mL TGF-β group, B = 10 ng/mL TGF-β group, C = ARB group, D = 1 mg/mL SK group, E = 2 mg/mL SK group, F = 4 mg/mL SK group). [file 12906_2020_3180_MOESM2_ESM.docx]

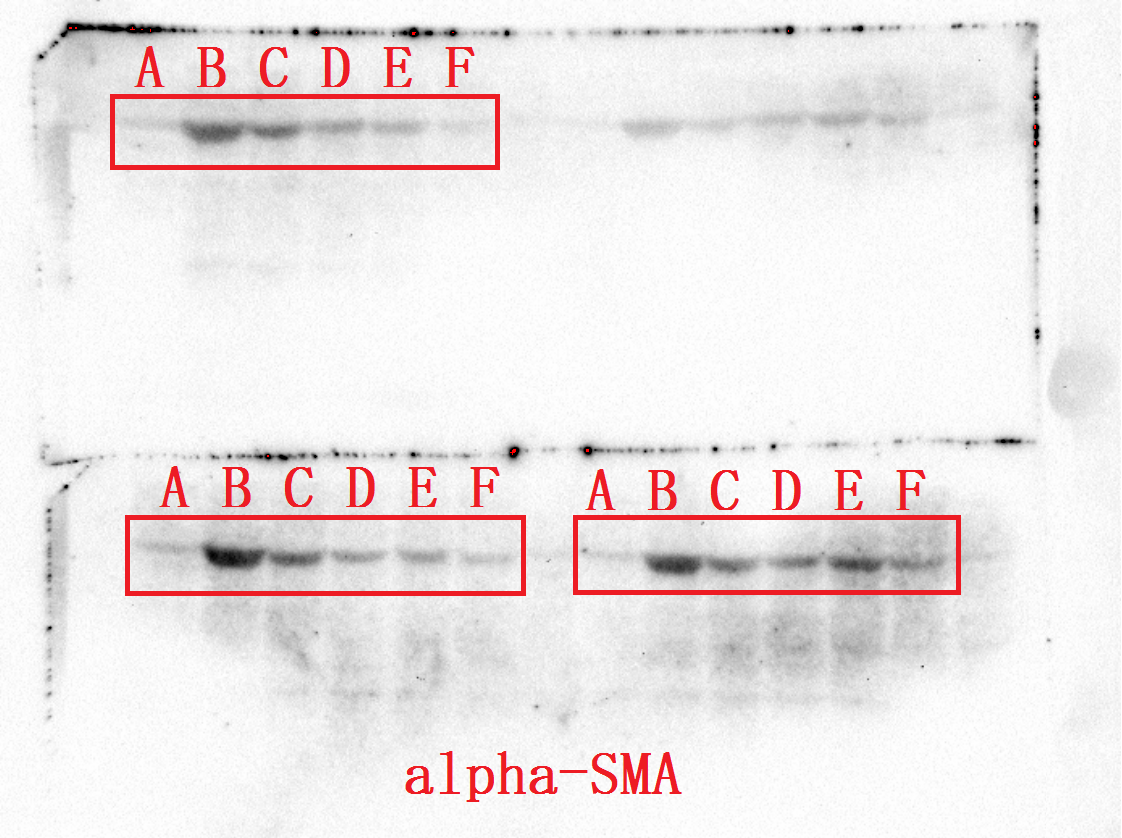


Figure 1: Expression of α-SMA in each group (A = 0 ng/mL TGF-β group, B = 10 ng/mL TGF-β group, C = ARB group, D = 1 mg/mL SK group, E = 2 mg/mL SK group, F = 4 mg/mL SK group).


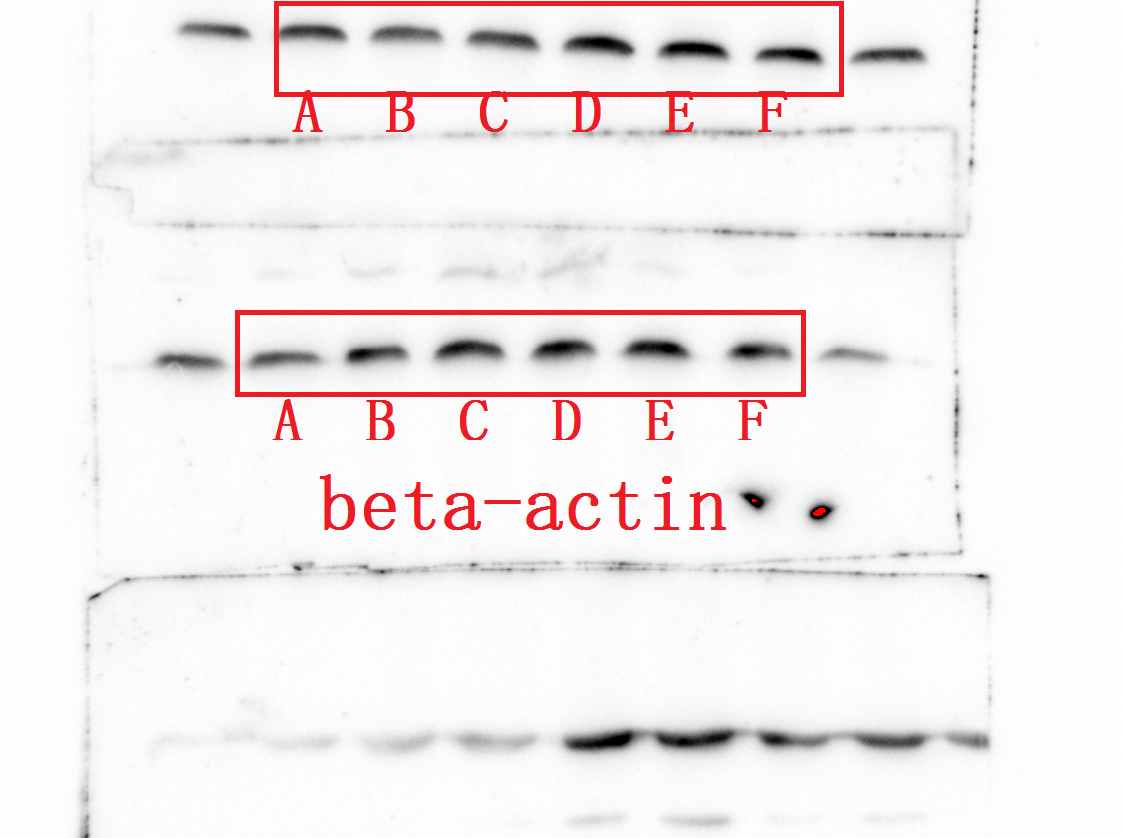


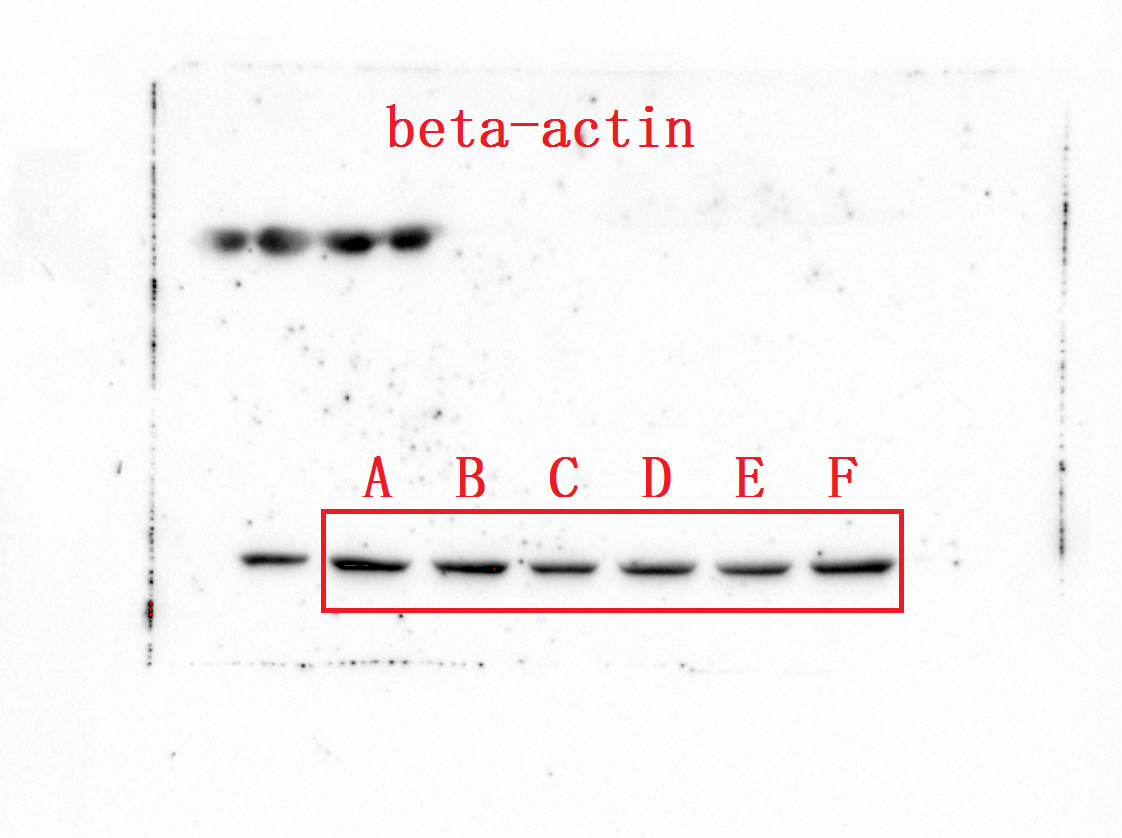


Figure 2: Expression of beta-actin in each group (A = 0 ng/mL TGF-β group, B = 10 ng/mL TGF-β group, C = ARB group, D = 1 mg/mL SK group, E = 2 mg/mL SK group, F = 4 mg/mL SK group).


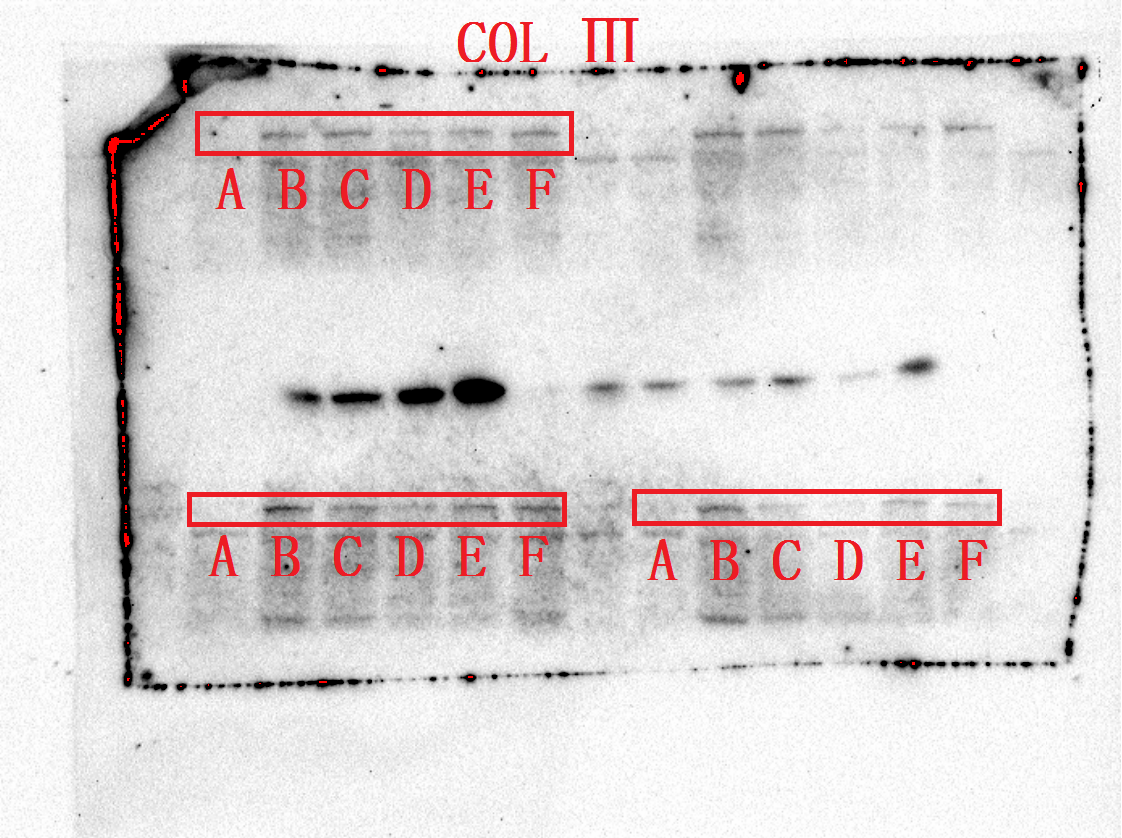


Figure 3: Expression of col Ⅲ in each group (A = 0 ng/mL TGF-β group, B = 10 ng/mL TGF-β group, C = ARB group, D = 1 mg/mL SK group, E = 2 mg/mL SK group, F = 4 mg/mL SK group).


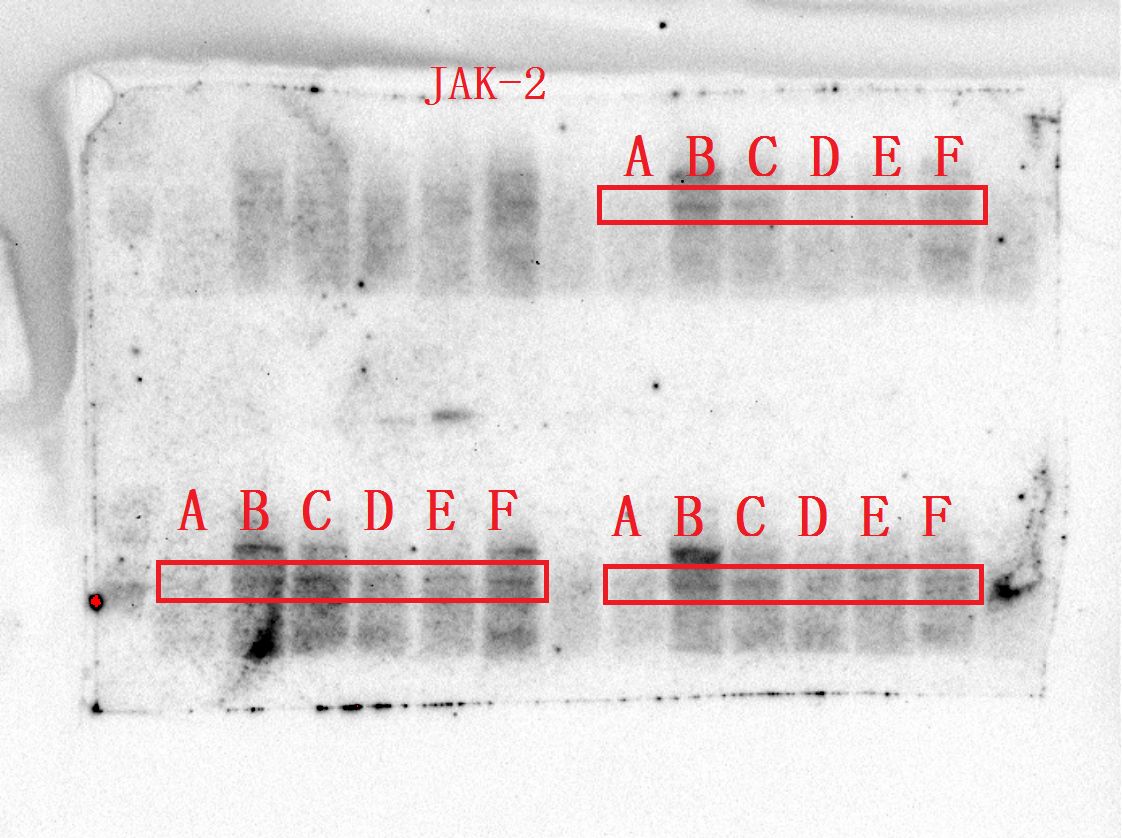


Figure 4: Expression of JAK2 in each group (A = 0 ng/mL TGF-β group, B = 10 ng/mL TGF-β group, C = ARB group, D = 1 mg/mL SK group, E = 2 mg/mL SK group, F = 4 mg/mL SK group).


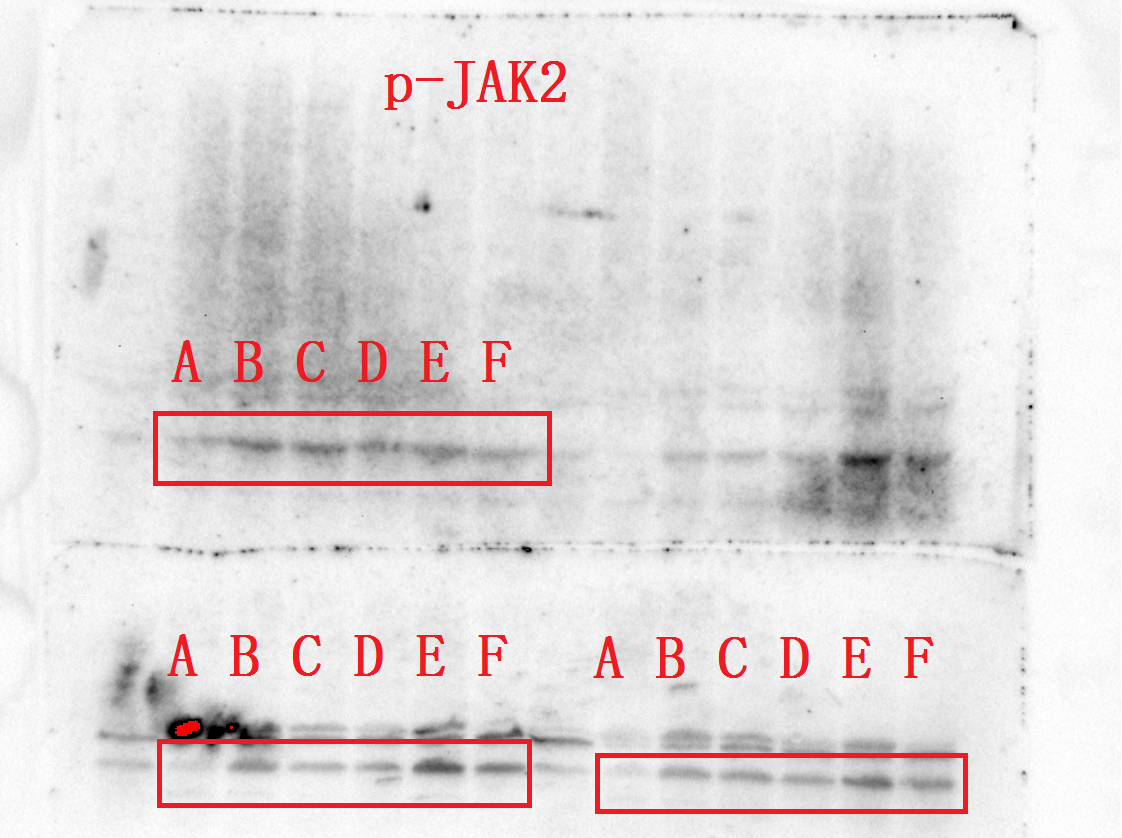


Figure 5: Expression of p-JAK2 in each group (A = 0 ng/mL TGF-β group, B = 10 ng/mL TGF-β group, C = ARB group, D = 1 mg/mL SK group, E = 2 mg/mL SK group, F = 4 mg/mL SK group).


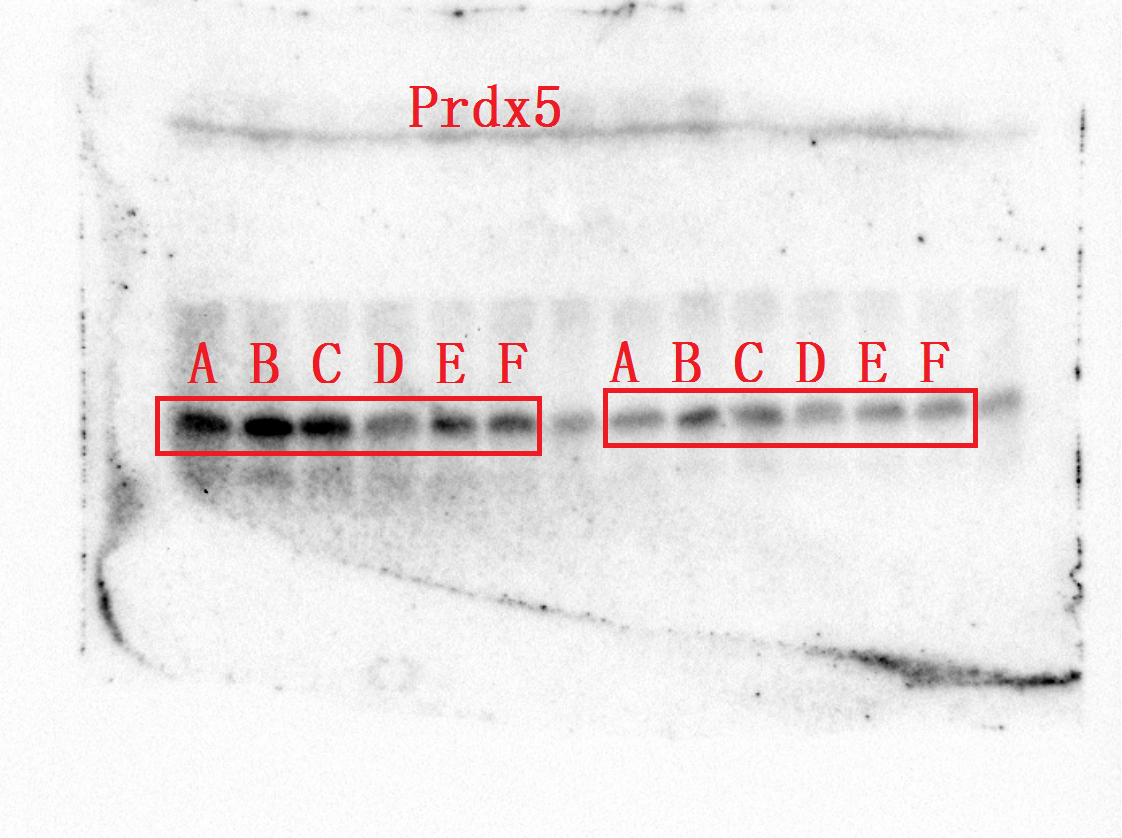


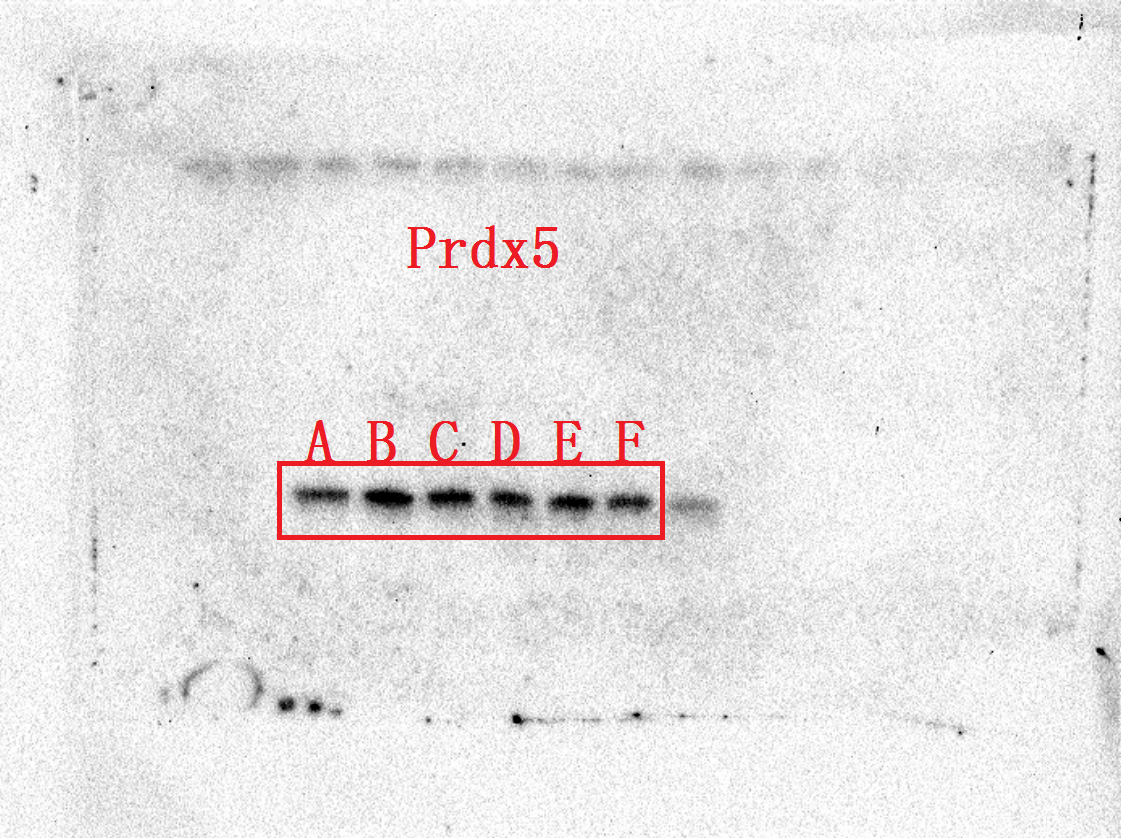


Figure 6: Expression of Prdx5 in each group (A = 0 ng/mL TGF-β group, B = 10 ng/mL TGF-β group, C = ARB group, D = 1 mg/mL SK group, E = 2 mg/mL SK group, F = 4 mg/mL SK group).


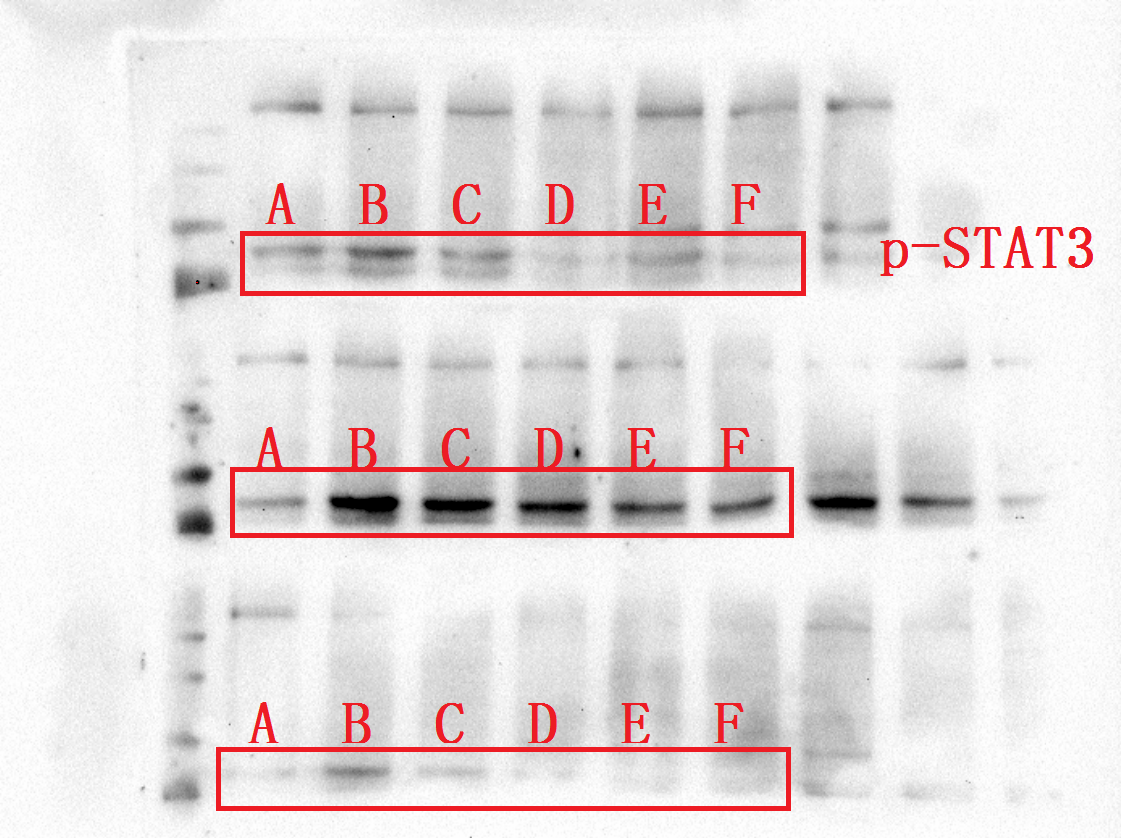


Figure 7: Expression of p-STAT3 in each group (A = 0 ng/mL TGF-β group, B = 10 ng/mL TGF-β group, C = ARB group, D = 1 mg/mL SK group, E = 2 mg/mL SK group, F = 4 mg/mL SK group).


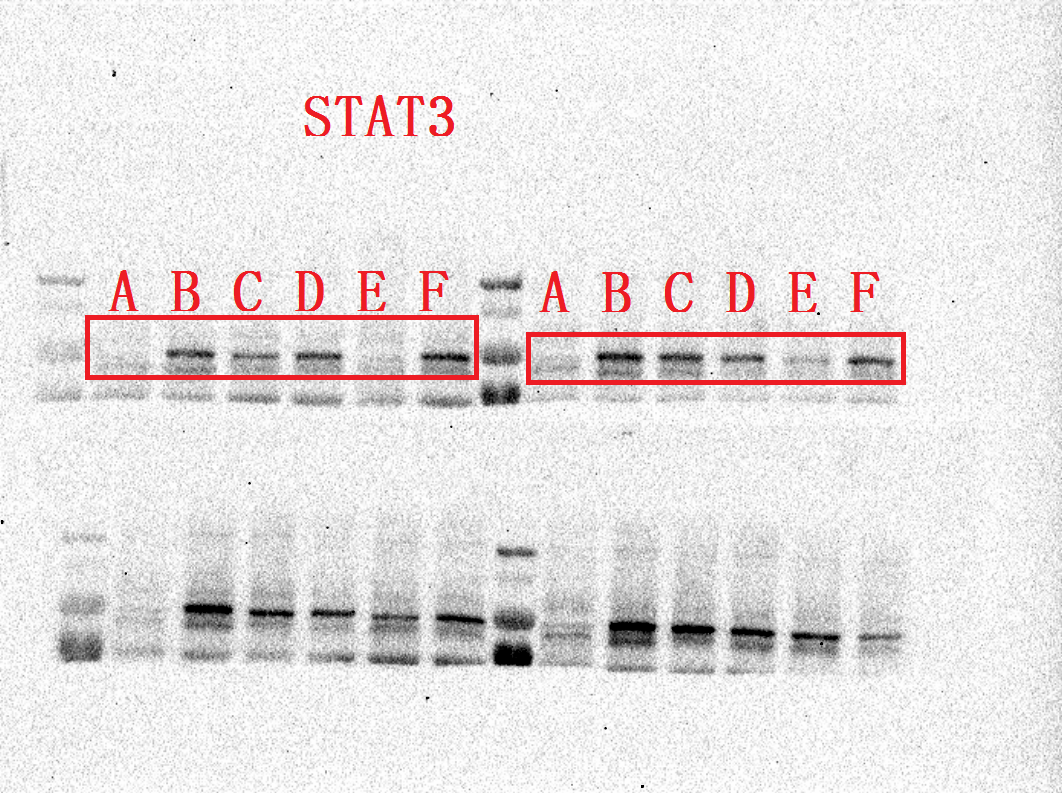


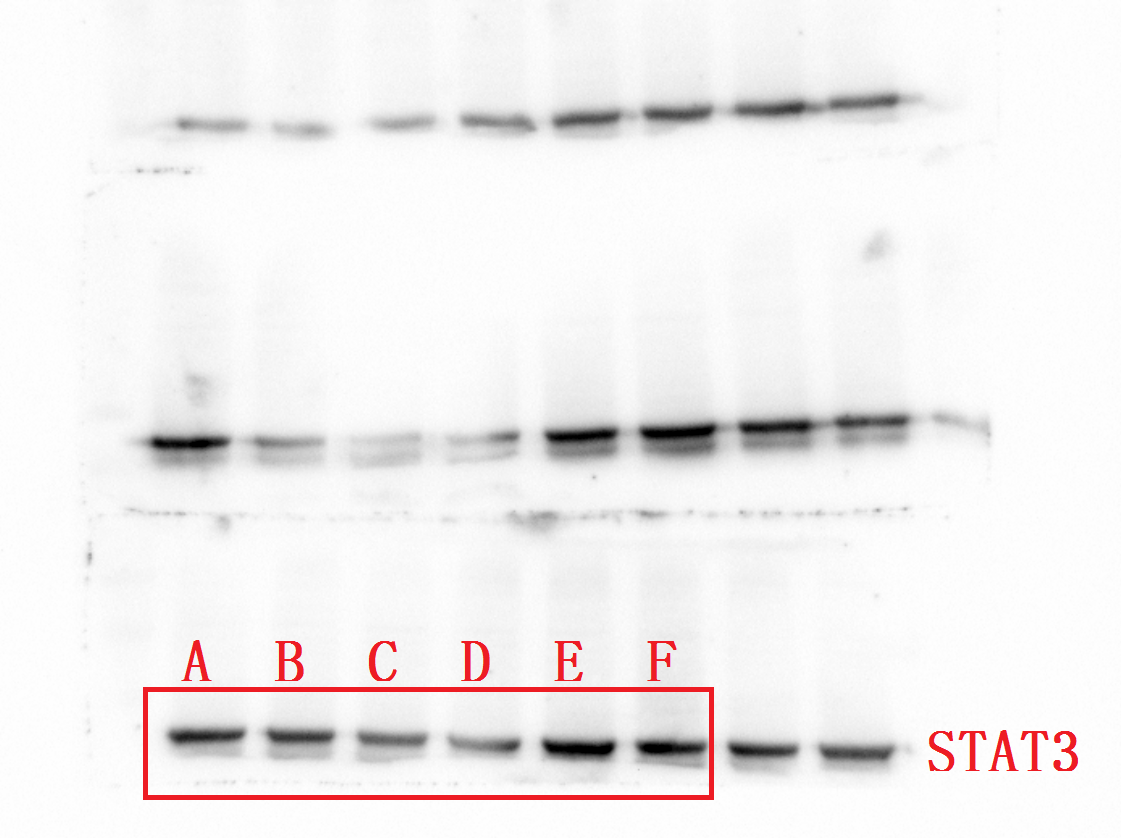


Figure 8: Expression of STAT3 in each group (A = 0 ng/mL TGF-β group, B = 10 ng/mL TGF-β group, C = ARB group, D = 1 mg/mL SK group, E = 2 mg/mL SK group, F = 4 mg/mL SK group).
